# Imbalanced sphingolipid signaling is maintained as a core proponent of a cancerous phenotype in spite of metabolic pressure and epigenetic drift

**DOI:** 10.18632/oncotarget.26533

**Published:** 2019-01-11

**Authors:** Monique M.P. Speirs, Adam C. Swensen, Tsz Y. Chan, Peter M. Jones, John C. Holman, McCall B. Harris, John A. Maschek, James E. Cox, Richard H. Carson, Jonathon T. Hill, Joshua L. Andersen, John T. Prince, John C. Price

**Affiliations:** ^1^ Department of Chemistry and Biochemistry, Brigham Young University, Provo, Utah, USA; ^2^ Health Sciences Cores-Metabolomics, University of Utah, Salt Lake, Utah, USA; ^3^ Department of Physiology and Developmental Biology, Brigham Young University, Provo, Utah, USA

**Keywords:** multi-omics analysis of cancer, clonal evolution, metabolic reprogramming, sphingolipid signaling, sphingosine kinase

## Abstract

Tumor heterogeneity may arise through genetic drift and environmentally driven clonal selection for metabolic fitness. This would promote subpopulations derived from single cancer cells that exhibit distinct phenotypes while conserving vital pro-survival pathways. We aimed to identify significant drivers of cell fitness in pancreatic adenocarcinoma (PDAC) creating subclones in different nutrient formulations to encourage differential metabolic reprogramming. The genetic and phenotypic expression profiles of each subclone were analyzed relative to a healthy control cell line (hTert-HPNE). The subclones exhibited distinct variations in protein expression and lipid metabolism. Relative to hTert-HPNE, PSN-1 subclones uniformly maintained modified sphingolipid signaling and specifically retained elevated sphingosine-1-phosphate (S1P) relative to C16 ceramide (C16 Cer) ratios. Each clone utilized a different perturbation to this pathway, but maintained this modified signaling to preserve cancerous phenotypes, such as rapid proliferation and defense against mitochondria-mediated apoptosis. Although the subclones were unique in their sensitivity, inhibition of S1P synthesis significantly reduced the ratio of S1P/C16 Cer, slowed cell proliferation, and enhanced sensitivity to apoptotic signals. This reliance on S1P signaling identifies this pathway as a promising drug-sensitizing target that may be used to eliminate cancerous cells consistently across uniquely reprogrammed PDAC clones.

## INTRODUCTION

Cancer development is a highly dynamic biochemical process driven by both neutral evolution and environmental pressure [1]. Due to the combined influences of stochastic and selective factors, like genetic instability and metabolic stress, a single originating cancer cell can give rise to heterogeneous clonal populations with distinct genetic and/or phenotypic profiles [2]. Inter- and intra-tumor heterogeneity promote drug resistance and limit the predictability of cancer prognosis [3–5]. Alternatively, multiple subclones may exhibit parallel evolution, whereby specific adaptations or pro-cancer pathways are selectively *maintained* throughout tumor progression [6]. Conserved pathways provide a degree of evolutionary predictability [3] and potentially serve as ubiquitous drug targets among heterogeneous cancer subclones [7, 8]. Predicting which pathways are retained so that different subclones will consistently respond to treatments, versus those which are frequently divergent, remains limited in most tumor types [3].

Pancreatic ductal adenocarcinomas (PDAC) display frequent, severe levels of inter- and intra-tumor heterogeneity driven by successive genetic and epigenetic modifications in early and metastatic stages [9]. Chemotherapy is effective in some patients, but most tumors develop resistance mechanisms and efforts to improve standard chemotherapeutic procedures have failed clinical trials [10]. An increased understanding of conserved pathways at the genomic, transcriptomic, and metabolic levels of PDAC cellular evolution will pave the way for novel therapeutic opportunities [9].

A growing body of work reveals that deregulation of lipid metabolism (both structural and signaling lipids, Supplementary Figure 1) may be one of the most definitive metabolic hallmarks of cancer, presenting important targets for therapeutic intervention [11–19]. Cancer-promoting changes in lipid utilization and signaling may be traced back to the core lipid-metabolizing enzymes [15, 16, 20–23]. Altered expression and/or regulation of lipid modifying enzymes can drive pro-cancer lipid metabolism and signaling. In many tumor types, mRNA and protein expression of Fatty Acid Synthase (FASN) are increased to fuel demands for *de novo* lipid synthesis to support new membrane formation and energy production [20, 24]. FASN and other lipid-modifying enzymes are involved in complex molecular networks including both signaling and non-effector metabolites with multiple points of interplay between complimentary and competing signals. Though many substrates within these networks are structurally similar, even small modifications to a given lipid can impose vastly different physiological effects [13].

**Figure 1:**
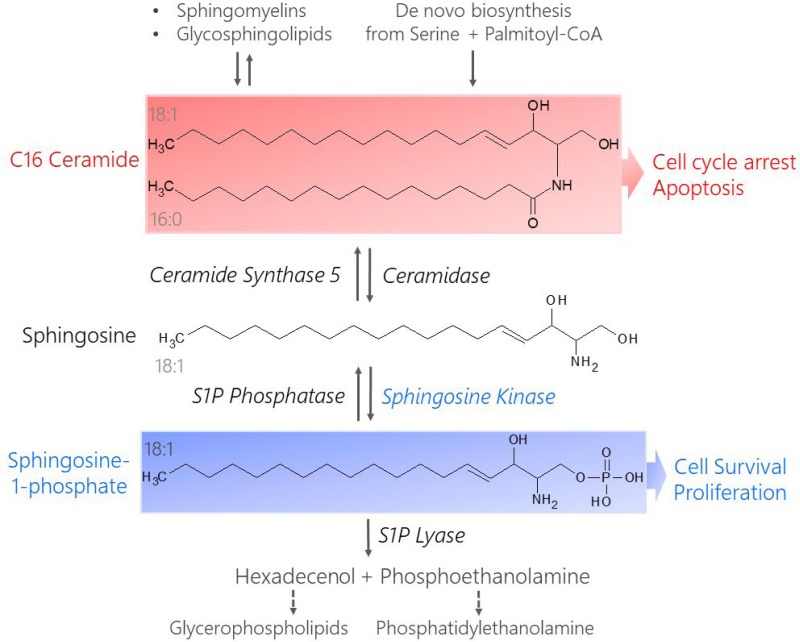
Structures and metabolism of pro-apoptotic C16 Cer and pro-survival S1P Ceramides result from the breakdown of more complex sphingolipids like sphingomyelins and glycosphingolipids or are synthesized *de novo* from serine and palmitoyl-CoA (C16 Cer shown). Ceramidase catalyzes the de-acylation of Ceramide to form sphingosine. Sphingosine Kinase phosphorylates sphingosine in an ATP-dependent manner to generate S1P. S1P is removed from the sphingolipid metabolism pathway when it is degraded by S1P Lyase, yielding precursors for phospholipid synthesis (hexadecenol and phosphoethanolamine). Ceramide can be recycled via S1P phosphatase-catalyzed dephosphorylation of S1P to reform sphingosine, which is acylated by Ceramide Synthase (CerS) to reform ceramide. The chain length of the resulting ceramide depends on the type of CerS that acts on sphingosine, e.g. CerS5 produces C16 Cer from sphingosine. C16 Cer promotes cell cycle arrest and apoptosis while S1P stimulates pro-survival and pro-proliferative signaling cascades.

Dysregulated signaling through bioactive sphingolipids shifts the balance between pro-growth versus pro-death pathways in cancer cells [11, 12, 25, 26]. Two interconvertible sphingolipid metabolites, ceramide and sphingosine-1-phosphate (LipidMaps ID# LMSP01050001, S1P), have been shown to have competing signaling roles in cancer cell fate [12, 27–30] (Figure 1). Ceramide is metabolized to form S1P in two enzymatic steps (deacylation and phosphorylation) by the protein Sphingosine Kinase (SK). At basal levels, ceramide is continuously recycled from S1P by the reverse of these two reactions. This ceramide salvage pathway can also be signal-mediated to alter endogenous ceramide concentrations relative to S1P in order to promote stress tolerance [30]. Current research indicates C16 Ceramide (LipidMaps ID# LMSP02010004, Cer(d18:1/16:0), Figure 1) is a potent pro-apoptotic signal involved in cell cycle arrest, cell senescence, and tumor suppression [31–36]. Alternatively, S1P acts as a pro-survival signal by promoting stress tolerance, cell motility, angiogenesis, and optimal growth factor induced proliferation [30, 33]. Although endogenous S1P is generally less abundant than ceramide, it is highly mobile and suppresses ceramide-induced apoptosis [37]. These findings by Cuvillier *et al.* led to the birth of the term “sphingolipid rheostat” which is used to describe the interplay between competing ceramide and S1P signals and their opposing effects on cell fate [30, 37].

While several enzymes are involved in the synthesis, degradation, and turnover of C16 Cer and S1P (Figure 1), literature suggests that Sphingosine Kinase 1 (SK1) plays a central role in regulating the sphingolipid rheostat [38–46]. Overexpression of SK1 has been reported in a wide range of tumors, including breast, colon, lung, ovarian, kidney, and rectal tumors [45]. Elevated SK1 activity is linked to tumor angiogenesis and progression as well as resistance to radiation and chemotherapy [45]. Therefore, SK1 may serve as a powerful drug target to shift the sphingolipid rheostat toward a healthy balance between pro- and anti-apoptotic signals in drug resistant cancers.

**Figure 2:**
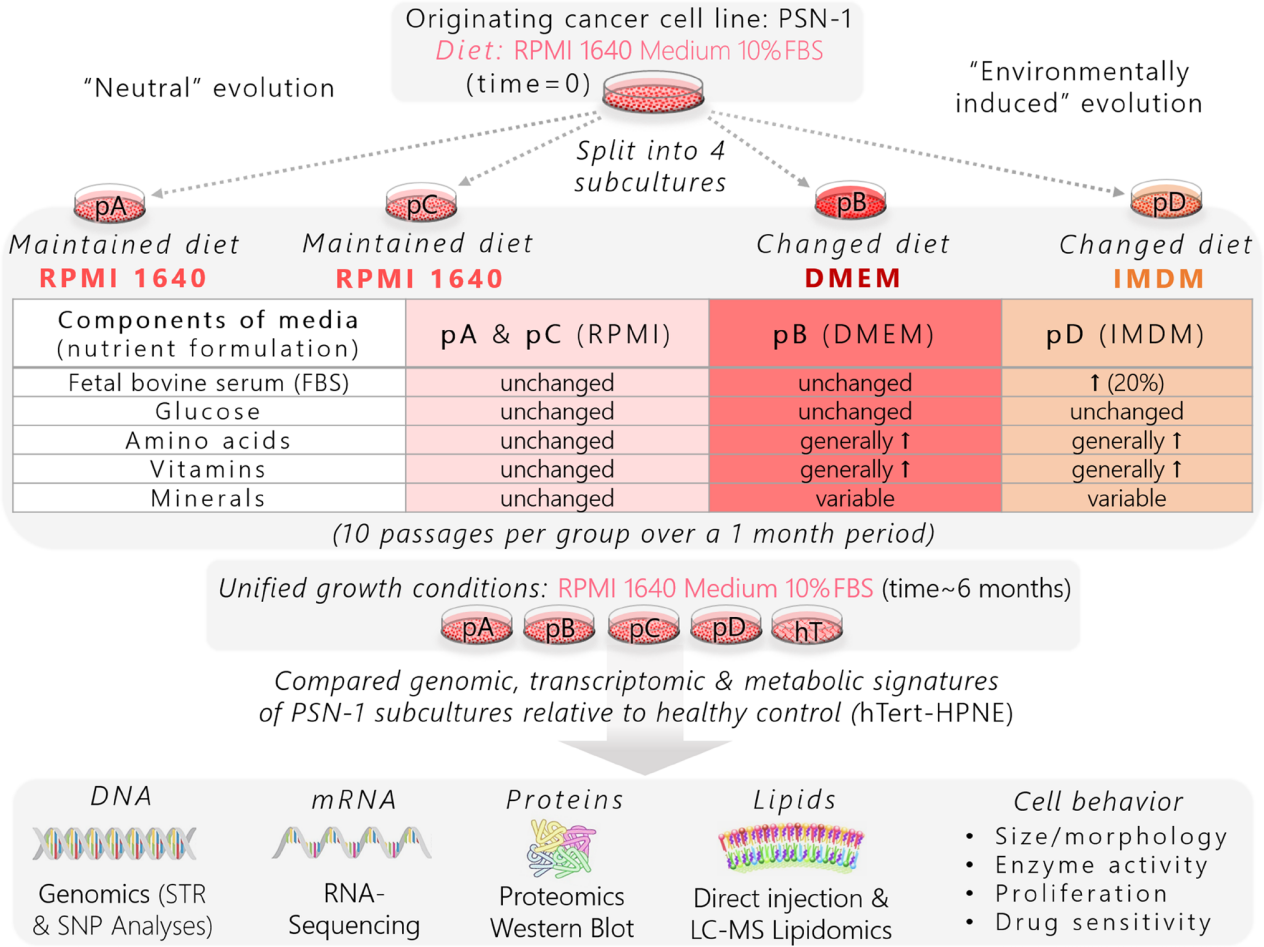
Schematic of experimental workflow used to generate isolated pancreatic cancer subclones from a common genetic origin and identify pro-survival pathways

Here, we sought to explore cancer cell evolution and identify conserved pathways among differentially evolved clonal populations that contribute to the aggressive and drug-resistant nature of PDAC. We developed a panel of phenotypically heterogeneous human PDAC cell populations from the same genetic origin (PSN-1) [47] to investigate how micoenvironmental pressures promote common and differential evolutionary paths in pancreatic cancer (Figure 2). The original PSN-1 stock was split into four isolated subcultures: psn1-A (pA), psn1-B (pB), psn1-C (pC), and psn1-D (pD). The pA and pC groups were passaged in standard growth conditions while pB and pD cells were subcultured using different nutrient formulations (Supplementary Figure 2) for one month. The pA and pC groups were used to represent a form of “neutral evolution” since they were influenced purely by internal stresses, such as rapid division rates, which have been shown to promote spontaneous genetic and metabolic instability [2]. In addition to “neutral” evolutionary stress, the pB and pD cells were introduced to modified microenvironmental cues from the new nutrient formulations, thereby representing subclones influenced both by stochastic internal and environmentally induced external pressures (Figure 2).

We compared the genetic and metabolic signatures of the four PSN-1 subclones to one another. We also used a non-oncogenic immortalized ductal pancreatic cell line, hTert-HPNE (hTert, hT) [48] as a healthy control in each assay to provide context for how much human ductal pancreatic cells can change their biochemistry and relative to the changes between individual subclones (Figure 2). Although our genomics data suggested the four PSN-1 subclones were virtually isogenic, they exhibited consistent *phenotypic* variations, suggesting that each cancer group followed a unique evolutionary path driven by non-genetic variations in molecular expression and regulation. At the same time, all four subclones maintained similar cancer-like phenotypes relative to hTert, such as irregular cell shapes and morphology, rapid proliferation rates, altered enzyme expression and activity levels, as well resistance to apoptotic signaling. This suggests that the most important pro-cancer pathways were selectively conserved across all four PSN-1 clones.

Despite numerous differentially expressed genes and metabolic modifications between individual subclones, each of our assays identified SK1-mediated S1P/C16 Cer metabolism as a key element regulating the shift between cancerous and healthy phenotypes in heterogeneous clonal populations. We propose that the selective pressure to maintain rapid growth and apoptotic resistance promotes this shift in SK1-mediated S1P/C16 Cer metabolism because it is a significant component of metabolic reprogramming in human pancreatic cancer cells. This “cancerous” sphingolipid rheostat is promoted through synergistic modification of transcription, translation, and enzyme activation, yet may be corrected in any subclonal variant through selective regulation of this metabolic pathway (Figure 1).

## RESULTS

### PDAC subclones and healthy controls displayed variations in cell size and morphology

Morphological phenotypes are intimately linked with shifts in cell stress, transcription, enzyme activity, and metabolism, thus serving as structural manifestations of interplay between environmental and intracellular cues [49]. Healthy control (hTert) cells were compared to the cancer subclones in order to establish a relevant size range. The hTert cells were extremely elongated with little to no rounded centers or terminal ends relative to the cancer groups (Supplementary Figure 3A–3E). All four cancer lines were significantly smaller than hTert cells (*P* < 0.01), which may be connected to their rapid cell division rates.

Within the cancer groups, each subclone displayed specific morphological characteristics. The pA subclones exhibited both punctate and spheroid cell shapes composed of very rounded centers with short, pursed edges (Supplementary Figure 3A). The pB cells were generally thinner, less defined, and more elongated with rigid, sharp corners and darker nuclei than the other cancer groups (Supplementary Figure 3B). The pC cells portrayed plumper, concave spindle shapes with both smooth and sharp edges (Supplementary Figure 3C). The pD group included very punctate as well as fusiform cell shapes with well-defined, smooth edges (Supplementary Figure 3D). On average, pD cells were larger than the other cancer subclones and this difference was significant between pA and pD groups (*P* = 0.005) (Supplementary Figure 3F). We hypothesized that these phenotypic variations may be indicators of biochemical perturbations between cancerous and healthy cells as well as between individual PSN-1 subclones.

### DNA fingerprints were identical in distinct PDAC clonal populations

STRs are short, tandemly repeated DNA sequences (~2–6 bp) scattered somewhat evenly throughout the human genome [50]. Because STRs display high degrees of polymorphism between individuals, they are used to produce a unique numerical pattern made up of 8 STR markers (along with amelogenin for sex determination) known as the “DNA fingerprint” (Supplementary Figure 4A–4B) [51]. A cell line is considered authentic when there is a ≥80% match between the sample cell line and the reference STR profile [51]. As a reference, there was a 100% match between our hTert cells and the American Type Culture Collection (ATCC) reference profile for hTert-HPNE (Supplementary Figure 4A).

We compared STR profiles of cells from each PSN-1 subclone (pA, pB, pC, pD) collected at the end of the study (time~6 months) to cells from the original PSN-1 stock (time = 0) (Figure 2). The original stock displayed a 92% match with the ATCC PSN-1 reference profile (Supplementary Figure 4B), indicating that our originating PSN-1 line was an authentic representation of the PSN-1 human cell line [47]. Each of the four PSN-1 subclones (pA, pB, pC, and pD) displayed equivalent matches (92%) with the PSN-1 reference profile. This indicates that any evolutionary changes that may have occurred throughout the study did not affect the DNA fingerprint nor the ability to trace each PSN-1 subclonal population back to the original tissue donor.

### PDAC subclones exhibited distinct nscSNP profiles relative to healthy control cells, but were virtually isogenic relative to one another

Single nucleotide polymorphisms (SNPs) resulting from selectively maintained point mutations are the most common type of genetic variation throughout the human genome [52]. Non-synonymous SNPs in coding regions (nscSNPs) and regulatory regions of the genome tend to have the greatest effects on phenotype [53] and may provide a foundation for cancer development and tumor heterogeneity [52, 54, 55]. Sequence variant analysis from RNA-Seq data was used to compare the genomes of each cell type (hT, pA, pB, pC, pD). There were numerous nscSNPs detected in each sample from both hTert and cancer groups relative to the reference genome (640,451 total nscSNPs detected in 13,657 total genes across 16 samples) (Supplementary Database 1). Interestingly, the median nscSNP density was highest in the slower growing hT cells (0.059%), followed by pC (0.049%), pA (0.044%), pB (0.043%), and pD cells (0.039%) (Supplementary Database 1). This indicates that the genomes of both cancer and healthy ductal pancreatic cell cultures were susceptible to nscSNP-driven genetic variation.

**Figure 3:**
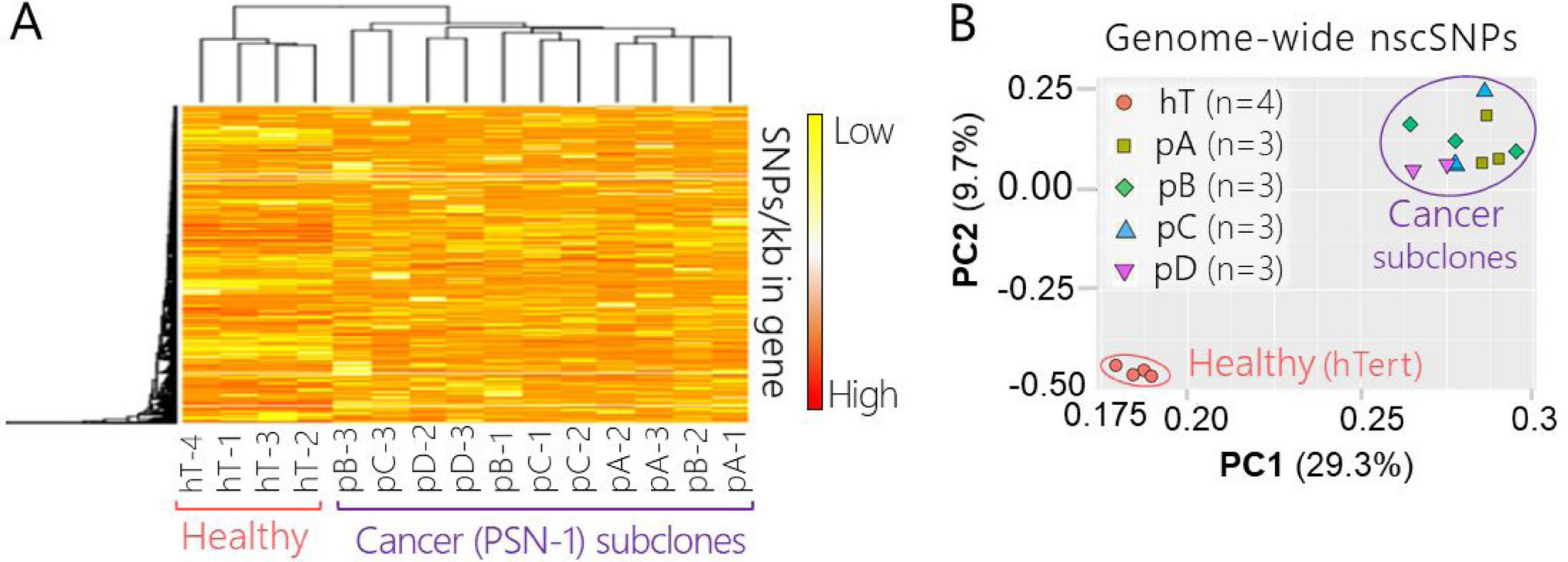
Genome-wide nscSNP Analysis PSN-1 subclones and healthy control cells (**A**) Hierarchal clustering and heat map of non-synonymous coding SNPs detected via RNA-Seq of healthy control cells (hT) and PSN-1 subclones (pA, pB, pC, pD) (*n* = 640,451 nscSNPs). Measurements were collected in biological triplicate or quadruplicate, (all 16 shown for comparison); the group name and replicate number are shown for each sample. Rows were centered; no scaling was applied to rows; both rows and columns were clustered using Hierarchal Euclidean distance metric with complete linkage. Each row represents a different gene (*n* = 13,657 genes). The scale from low (yellow) to high (red) represents the relative level of nscSNPs normalized to the gene length (kb) that were detected in the respective sample. (**B**) PCA of nscSNPs measured via RNA-Seq of healthy control cells (hT) and PSN-1 subclones (pA, pB, pC, pD) (*n* = 640,451 nscSNPs). Measurements were collected in biological triplicate or quadruplicate, resulting in a total of 16 samples. SVD was used to calculate principal components; X and Y axis show principal component 1 and principal component 2, which explain 29.3% and 9.7% of the total variance, respectively. The orange circles represent hT samples, green squares represent pA samples, bright green diamonds represent pB samples, blue triangles represent pC samples, and purple upside-down triangles represent pD samples. The pink open circle is shown to differentiate samples from the healthy control group (hT) from those of the cancer subculture groups (pA, pB, pC, pD) which are clustered within the purple circle. One biological replicate from the pC group and one from pD group are not visible due to overlap with points from biological replicates of the same group.

We used heat map clustering to visualize broad differences between gene specific nscSNP densities in each sample (Figure 3A). All four hT biological replicates clustered together (left) and did not intermix or cluster with nscSNP profiles from any cancer sample (right) (Figure 3A). On the other hand, nscSNP profiles of biological replicates from the four cancer groups were quite intermixed and clustered together throughout the right-hand portion of the heat map (Figure 3A). Overall, there were no clear differences distinguishing the genome-wide nscSNP signatures of the four PSN-1 subclones relative to one another (Figure 3A).

We used a Principle Component Analysis (PCA) of the SNP data in binary form (presence or absence of a nscSNP) to investigate genome-wide differences in these coding region SNPs between each group (Figure 3B). There was a clear separation between the nscSNP profiles of all four hTert replicates relative to the cancer samples, suggesting that there were a large number of differences in nscSNP densities per gene between the healthy and cancer groups (Figure 3B). On the other hand, all of the biological replicates derived from the four different PSN-1 clones (3 from each group) formed another fairly isolated cluster on the PCA plot (Figure 3B). This further indicates that the genome-wide nscSNPs densities between the different cancer groups did not significantly change throughout the subculturing experiment. The overlapping points of the subclone replicates (Figure 3A) suggest that there were no significant differences in global nscSNP compositions between individual PSN-1 subclones.

### Isogenic PDAC subclones displayed significant variations in global mRNA expression

We compared the relative mRNA concentrations to identify genes that were differentially expressed between cell types using the *DESeq2* Bioconductor package statistical criteria [56]. There were 19,946 common genes quantified in all of the groups that met the statistical criteria for quantitative mRNA analysis (See Materials and Methods) [56]. Relative to healthy (hT, pA, pB, pC, pD) (Supplementary Database 2), there were about half as many significant differentially expressed genes between the cancer subclones (31.3%; adjusted *P* < 0.1) (Supplementary Database 3).

**Figure 4:**
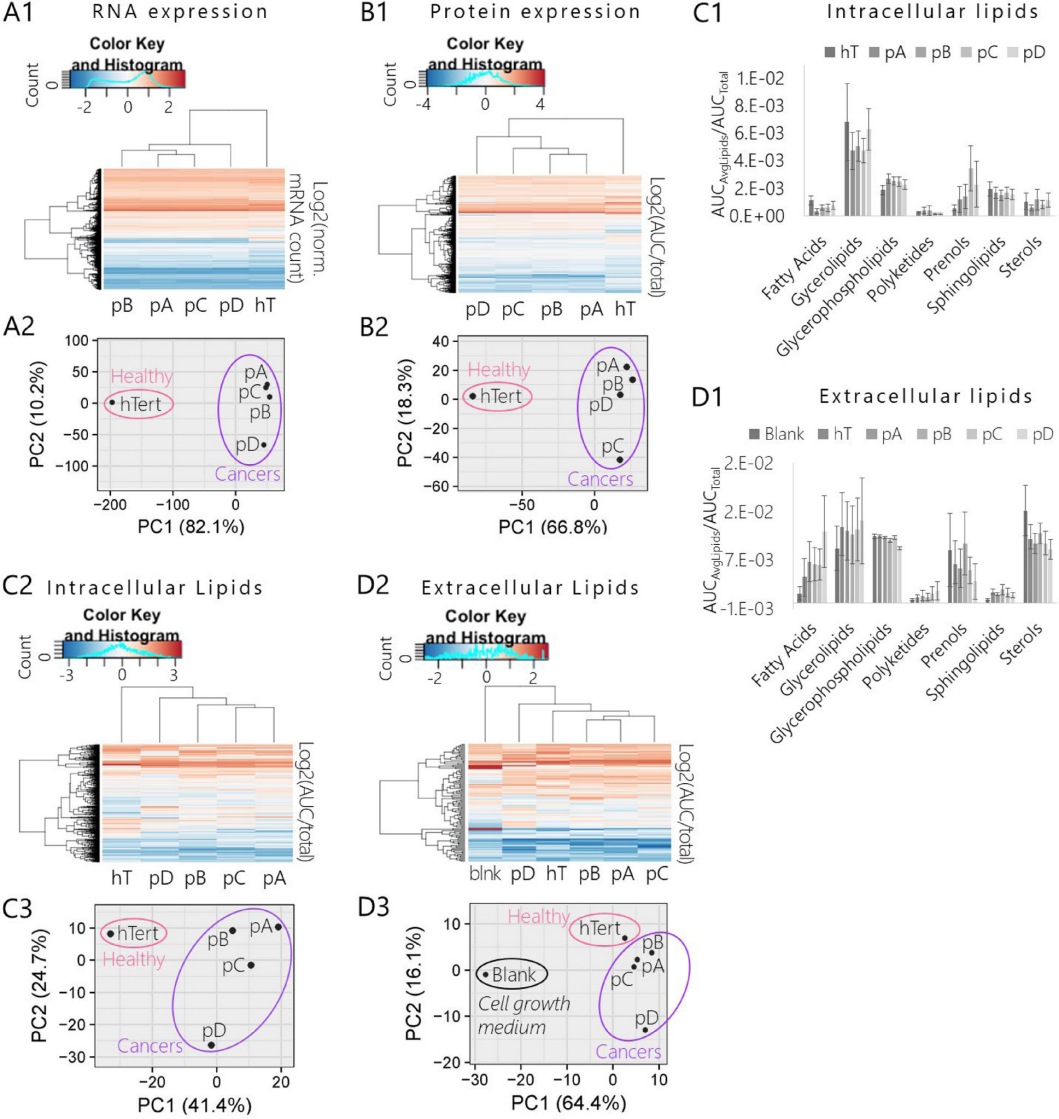
Comparison of mRNA, protein, and lipid expression profiles of pancreatic cancer subclones (pA, pB, pC, pD) and healthy control (hT) (**A1-A2**) Results of RNA-Seq assay of global mRNA extracted from pancreatic cancer subclones and healthy control cell lysates. (**A1**) Heat map and hierarchal clustering of cancer subclone and hT transcriptomes. Rows were centered; no scaling was applied to rows; both rows and columns were clustered using Hierarchal Euclidean distance metric with complete linkage. Each row represents a unique gene (*n* = 19,946 genes). The color scale from -2 (blue) to 2 (orange) represents the mean normalized mRNA concentration of 3-4 biological replicates per group (3 per cancer group and 4 hT) calculated for each gene as Log2(normalized mRNA Counts of gene). (**A2**) PCA of cancer subclone transcriptomes and healthy control. No scaling was applied to rows; SVD with imputation was used to calculate principal components; X and Y axis show principal component 1 and principal component 2 that explain 82.1% and 10.2% of the total variance, respectively. (**B1-B2**) Results of global quantitative proteomics analysis of proteins extracted from whole cell lysates of pancreatic cancer subclones and hT cultures. (**B1**) Heat map and hierarchal clustering of cancer subclone and healthy control global proteomes. Rows were centered; no scaling was applied to rows; both rows and columns were clustered using Hierarchal Euclidean distance metric with complete linkage. Each row represents a unique protein identification (*n* = 1,378 proteins). The color scale from -4 (blue) to 4 (orange) represents the mean normalized protein concentration of 3–4 biological replicates per group (4 per cancer group and 3 hT) calculated for each protein as Log2(AUC_Protein_/AUC_Total_). (**B2**) PCA of cancer subclone and healthy control cell proteomes. No scaling was applied to rows; SVD with imputation was used to calculate principal components; X and Y axis show that principal component 1 and principal component 2 explain 66.8% and 18.3% of the total variance, respectively. (**C1-C3**) Results of quantitative lipidomics analysis measured by LC-MS of lipids extracted from pancreatic cancer subclones and hT cell lysates. (**C1**) Heat map and hierarchal clustering of cancer subclone and healthy control intracellular lipidomes. Rows were centered; no scaling was applied to rows. Both rows and columns were clustered using Hierarchal Euclidean distance metric with complete linkage. Each row represents a unique lipid annotation (*n* = 500 species). The color scale from -3 (blue) to 3 (orange) represents the mean normalized lipid concentration of three biological replicates per group calculated for each lipid as Log2(AUC_Lipid_/AUC_Total_). (**C2**) Categorized intracellular lipidome compositions of species measured via quantitative intracellular LC-MS analysis. Data are expressed as the mean (Avg) normalized concentration (AUC_AvgLipids_/AUC_Total_) of total lipids measured in each category ± SEM measured in three biological replicates per cell type. (**C3**) PCA of cancer subclone and healthy control intracellular lipidomes. No scaling was applied to rows; SVD with imputation was used to calculate principal components; X and Y axis show principal component 1 and principal component 2 that explain 41.4% and 24.7% of the total variance, respectively. (**D1-D3**) Results of quantitative extracellular lipidomics analysis measured by LC-MS of lipids extracted from the complete growth medium used in cell cultures during intracellular lipidomics experiment (RPMI 1640 + 10% FBS). The negative control or “Blank” (blnk) represents the lipidome of fresh complete cell medium that was never exposed to cell cultures. (**D1**) Hierarchal clustering and heat map of cancer subclone and healthy control extracellular lipidomes. Rows were centered; no scaling was applied to rows. Both rows and columns were clustered using Hierarchal Euclidean distance metric with complete linkage. Each row represents a unique lipid annotation (*n* = 112 lipids). The color scale from -2 (blue) to 2 (orange) represents the mean normalized lipid concentration of 3 biological replicates per group calculated for each lipid as Log2(AUC of lipid/Sum AUC of total lipids quantified in sample). Rows were centered; unit variance scaling was applied to rows. Both rows and columns were clustered using correlation distance and average linkage. (**D2**) Categorized intracellular lipidome compositions of species measured via quantitative extracellular LC-MS analysis. Data are expressed as the mean (Avg) normalized concentration (AUC_AvgLipids_/AUC_Total_) of total lipids measured in each category ±SEM measured in three biological replicates per cell type. (**D3**) PCA of cancer subclones and healthy control extracellular lipidomes compared to blank. No scaling was applied to rows; SVD with imputation was used to calculate principal components. X and Y axis show principal component 1 and principal component 2 that explain 64.4% and 16.1% of the total variance, respectively.

Hierarchal heat map clustering and a PCA were used to explore general differences in the global mRNA expression profiles (*n* = 19,946 genes) of each group (Figure 4A1-A2). Both the heat map (Figure 4A1) and PCA (Figure 4A2) displayed a very clear division between mRNA profiles of the cancer groups (pA, pB, pC, pD) relative to the healthy control (hT). There were also fewer significant differences in mRNA expression levels between the different PSN-1 subclones (Supplementary Database 3), demonstrating that the cancer groups altered transcription in different ways over the course of the subculturing experiment. Based on the PCA plot (Figure 4A2), the clones most similar in terms of mRNA expression were those whose nutrient formulations were unchanged (pA and pC) (Figure 2). In contrast, cancers subcultured in different nutrient formulations (pB and pD) displayed greater degrees of variance relative to pA and pC. Subclones subjected to the more extreme nutrient change (pD, wherein FBS was increased two-fold) displayed the greatest variation in global mRNA levels relative to the other three cancer subclones (pA, pB, pC) (Figure 4A2). This suggests that PSN-1 subclones not only altered mRNA expression levels in response to intrinsic sources of stress such as rapid cell division (as in the cases of pA and pC), but may have also further modified transcription in response to specific nutrient stresses, as exemplified by the pD cells.

### Isogenic PDAC subclones displayed global shifts in protein expression

To determine whether these global shifts in transcription affected the proteome, we performed a comparative quantitative proteomics analysis of each cell group. There were 1,378 unique proteins identified across all groups (Supplementary Database 4) that met our statistical criteria for quantitative analysis and protein expression profiling. We used hierarchal heat map clustering and a PCA to compare the protein expression profiles of each cell type (Figure 1–2). Similar to our RNA-Seq data (Figure 4A1–A2), the heat map and PCA of our proteomics data revealed a very clear separation between the global protein expression profiles of the four cancer groups relative to the healthy control (Figure 4B1-2). Within the fairly tight cluster of points representing cancerous protein signatures on the PCA plot, the pC cells were measurably distant from the other three subclones (Figure 4B2). This suggests that protein metabolism was most altered in pC cells as a result of internal (non-environmental) pressures over the course of the subculturing process.

We performed a protein ontology analysis using David Bioinformatics Functional Annotation Tools [17] to determine whether certain types of functional proteins were differentially expressed between cell types. Among numerous significantly differentially expressed proteins in the hTert cells relative to the cancerous groups (corrected *P* value <0.05), ~39% were upregulated (fold change >1) and 61% were downregulated (fold change <1) (Supplementary Database 5). We were not surprised to find that the top three significantly enriched ontologies (Benjamini score for enrichment <0.05) among proteins that were significantly upregulated in hT relative to the cancer groups were involved in actin filament binding, focal adhesion, and cytoskeleton. A decrease in actin proteins and other cytoskeletal components may have accompanied the structural framework of the smaller, more punctate cancer cells relative to the longer, more elongated hT cells (Supplementary Figure 3). Among the list of significantly downregulated proteins in hTert relative to the cancer groups, the top three significantly enriched ontologies (Benjamini score for enrichment <0.05) were mRNA processing, mRNA splicing, and the spliceosome as a whole. This suggests that the cancer cells transcribe and process mRNA differently than hTert, which may have contributed to the severe global shift in the mRNA expression profiles of the cancer subgroups relative to hTert (Figure 4A2).

There was also some evidence for differential expression of specific protein ontologies between individual cancer subclones (Supplementary Databases 6–9). Neither pA nor pD cells displayed significant changes in recognized ontologies relative to the other subclones. The pB cells were upregulated in nucleotide binding, ATP-binding, and ATP-dependent RNA helicase activity. This suggests a difference in the way pB cells bind and use ATP, especially with regards to RNA processing, which may have contributed to variations in the global mRNA profile of pB relative to other subclones (Figure 4A1-2). The pC cells were upregulated in amino acid transport and metabolism proteins, the extracellular exosome, and metal-binding proteins. Altered expression of amino acid metabolism networks may have affected protein metabolism and contributed to the wide separation between the pC proteome relative to the other cancer subclones depicted on the PCA plot (Figure 4B2). Among the downregulated proteins in pC, the most enriched functional groups were translation and structural components of the ribosome. These results coupled with the evidence that proteins involved in amino acid metabolism were upregulated suggest that, compared to the other cancer groups, pC significantly altered protein synthesis and metabolism systems relative to the global proteome.

Overall, our protein ontology analysis showed that each PSN-1 subclone modified protein expression levels in unique ways to support cellular metabolism and morphology. However, we did not find any clear evidence of significant cell-fate signaling mechanisms that were conserved across the cancer subclones relative to the hTert. Because lipid metabolism and signaling play important roles in cancer cell fate (Supplementary Figure 1) we next explored methods to identify and monitor intracellular lipid concentrations.

### Isogenic PDAC subclones displayed global shifts in lipid concentrations including bioactive sphingolipids Sphingosine-1-phopshate and C16 Ceramide

We measured lipidomic profiles of each cell group in multiple experimental stages (Figure 4C, 4D, Supplementary Figure 5). To avoid background contamination from plastics, all lipid samples were prepared using glass pipettes and vials (See Materials and Methods). In our initial shotgun lipidomics assay using direct injection electrospray ionization mass spectrometry, a total of 980 unique lipids were identified among all five groups (Supplementary Database 10) that met our statistical criteria (See Materials and Methods). All eight lipid categories were represented in this list, including saccharolipids (0.4%), sterols (5.1%), polyketides (5.4%), prenols (7.2%), sphingolipids (10.0%), fatty acids (19.2%), glycerolipids (21.8%), and glycerophospholipids (30.9%). Relative concentrations of the detected lipids were used to develop preliminary lipidomic profiles of each cell type (Supplementary Figure 5).

The random stress clones (pA and pC) provide a baseline for changes due to rapid proliferation and extended culture. These clones were maintained on an unchanging nutrient formulation except that multiple batches of fetal bovine serum (FBS) were utilized during the month of culture. FBS is a natural product whose composition can change from batch to batch. Lipid extractions from RPMI 1640 cell culture medium containing three different batches of FBS (RPMI 1640 + 10% FBS) showed that concentrations of individual serum components can vary approximately 70% between batches (Median CV of lipid concentrations across three batches = 0.73, Supplementary Database 15). Based on paired *t*-tests comparing individual lipids across the different batches of FBS, there was no significant change overall across 800 lipid species quantified (*P* > 0.05, Supplementary Database 15). Changes in serum metabolite concentrations may have influenced random shifts in metabolic signatures between cell types. We attempted to control for these random changes by preparing cell culture medium for each group using the same batch of FBS throughout the study so that each subclone was affected by the same fluctuating serum-based variations in growth medium while pB and pD cells were also exposed to the designed nutrient changes (Figure 2).

Hierarchal heat map clustering and a PCA (Figure 4) illustrated a clear lipidome fingerprinting separation between cancer and healthy cells. This suggests that lipid concentrations and lipid metabolic networks were severely altered in relatively similar manners across the PDAC subclones compared to the healthy control. At the same time, both the heat map and PCA (Figure 4, Supplementary Figure 5) suggested apparent differences in global lipid levels between the four cancer groups, indicating that each subclone altered lipid expression and/or metabolism at slightly different degrees relative to one another during the subculturing experiment.

Most interestingly, our shotgun lipidomics analysis revealed connected differences between two interconvertible sphingolipid metabolites (Figure 1), C16 Cer (Supplementary Figure 5B) and S1P (Supplementary Figure 5C), in each subclone relative to hTert. C16 Cer levels were *depleted* in all four cancer groups relative to hTert and this decrease was significant in pB, pC, and pD cells (*P* < 0.05). On the other hand, S1P levels were *elevated* in all the cancer groups relative to hT and this increase was statistically significant in pA cells (*p* < 0.05). These preliminary results suggested that S1P production from C16 Cer (Figure 1) was suppressed in hTert cells whereas C16 Cer metabolism to S1P was upregulated to some degree in each cancer subclone. Despite numerous concentration differences throughout the lipidome between the different PSN-1 clones (Supplementary Figure 5A1-A2), these data suggested that these modified C16 Cer and S1P levels were conserved in the same direction across all four isolated PDAC clones relative to healthy control. Although C16 Cer and S1P levels were shifted by fairly different degrees in each subclone (Supplementary Figure 5B, 5C), PSN-1 cells may depend on some form of sphingolipid metabolite imbalance to regulate pro-survival pathways throughout different stages of progression or evolution. Our next goal was to verify these results and confirm whether this imbalance in the S1P/C16 Cer axis was indeed maintained as a pro-cancer mechanism among the PSN-1 subclones.

### Sphingolipid focused LC-MS confirmed that both global lipid expression and S1P/C16 Cer metabolism were modified in PDAC subclones relative to the healthy control

Although we consistently detected C16 Cer among hundreds of other lipid species, our initial direct injection lipidomics method was limited in its ability to consistently and accurately identify S1P species. Adapting the S1P focused LC-MS/MS approach developed by Bode and Gräler (B&G) [57], we measured S1P and C16 Cer along with the global lipidome of the PSN-1 subclones and hTert cells (Supplementary Database 4). The LC-MS/MS improved the confidence level of each lipid annotation using retention time (RT) (Supplementary Figure 6) and fragmentation alignment relative to deuterated internal standards (Supplementary Figures 7, 8).

A total of 500 lipids identified across all samples (*n* = 30) were used for quantitative lipidomic profiling after meeting our statistical criteria (see Materials and Methods) (Figure 4C1-3; Supplementary Database 11). A wide range of lipid species were represented in this list including glycerophospholipids (32.0%), sphingolipids (30.4%), sterols (13.6%), fatty acids (12.8%), polyketides (7.2%), glycerolipids (3.2%), and prenols (0.8%) at various levels of expression across the different cell types (Figure 4C1). The heat map (Figure 4C2) and PCA (Figure 4C3) of these data displayed similar trends between groups as observed in our initial shotgun assay (Supplementary Figure 5A1-2) despite the major changes in sample preparation, LC method, and MS instrument type that were used to produce the two sets of data.

There were significant degrees of variance between cancers, suggesting that each PSN-1 clone rerouted lipid metabolic pathways in different manners during the subculturing experiment. For example, as observed in the mRNA profiling analysis (Figure 4A1-A2), cells subjected to the most extreme nutrient formulation change (pD) displayed the greatest degree of variance in lipid expression relative to the other three cancer groups in the PCA (Figure 4C3). The concentrations of 16% of the quantified lipids were significantly different (corrected *P* < 0.05) in pD cells relative to the other three cancer groups (Supplementary Database 11). Note that pD cells were maintained in double the concentration of FBS (20%) as the other groups (10%) (Figure 2) which is the main source of available lipids in cell culture [58]. Among lipids that were significantly differentially expressed in pD cells (*P* < 0.05), 70% were reduced on average relative to the other cancer groups (fold change <1). This may suggest that the pD cells became dependent on the more abundant supply of lipids so that when they returned to the base media for the lipidomics experiment, intracellular lipid concentrations readily dropped compared to the other subclones that were fully accustomed to 10% FBS in the culture media. Overall, our global lipidomics data suggests PSN-1 cells alter global lipid metabolism in response to changes in microenvironmental resources.

**Figure 5:**
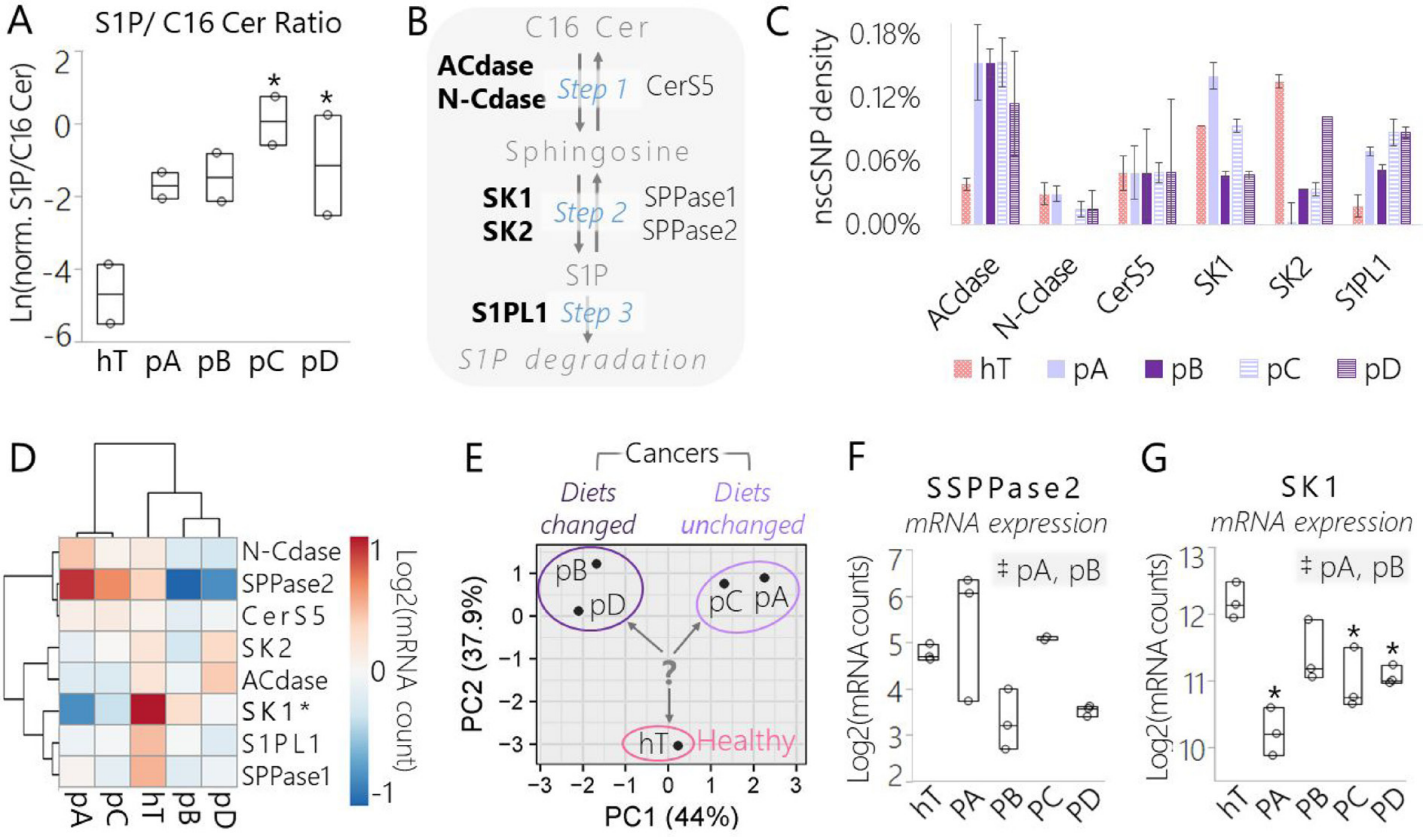
Data derived from lipidomics and RNA-Seq assays suggest a conserved shift in signaling sphingolipid metabolism in pancreatic cancer subclones relative to the healthy control driven in part by SK1 (**A**) Box plot of the normalized concentration of S1P over C16 Cer measured by shotgun lipidomics assay in each cancer subclone and hT whole cell lysates. Both sphingolipids were observed within the mass accuracy cutoff in 2 biological duplicates per group and the data are represented as the log transformed ratio of the normalized (norm.) concentrations of each lipid: Ln[(AUC_S1P_/AUC_Total_)/(AUC_C16Cer_/AUC_Total_)]. (**B**) Enzymes directly involved in C16 Cer/S1P metabolism identified by RNA-Seq of cancer subclones (pA, pB, pC, pD) and hT cell lysates. Enzyme names that are bolded displayed nonsynonymous coding SNP(s) in one or more of the cell types. (**C**) Densities of nscSNPs detected in one or more samples from each group. Data are represented as the number of nscSNPs normalized to the corresponding mRNA transcript length in kb. Error bars represent the SEM of 3-4 biological replicates per group. (**D**) Hierarchal clustering and heat map of mRNA levels of the enzymes that participate in the S1P/C16 Cer metabolism pathway shown in panel (B) measured by RNA-Seq of cancer clones (pA-D) and hT cell lysates. Rows were centered; no scaling was applied to rows; both rows and columns were clustered using Hierarchal Euclidean distance metric with complete linkage. The color scale from -1 (blue) to 1 (orange) represents the mean normalized mRNA concentration of 3–4 biological replicates per group on a Log2 scale. (**E**) PCA of mRNA levels of the enzymes involved in S1P/C16 Cer metabolism shown in panel (B) that were detected by RNA-Seq of cancer clones and hT cell lysates. No scaling was applied to data; SVD with imputation was used to calculate principal components; X and Y axis show principal component 1 and principal component 2 that explain 44% and 37.9% of the total variance, respectively. The question mark represents the major question that arose from this analysis: which enzyme(s) were important drivers of PC2, separating the healthy control from the cancer groups? (**F**–**G**) Box plots of normalized (F) SPPase2 and (**G**) SK1 mRNA levels measured by RNA-Seq of cancer subclones and hT cells. Data are represented as the Log2 transformed normalized mRNA counts measured in biological triplicate or quadruplet. (A, F–G) The ^*^indicates *P* < 0.05 with the Dunnett's Test used to compare measurements from all cancer groups to the healthy control. Tukey-Kramer Tests were used to determine significant differences between cancer subcultures; pairs that were significantly different are highlighted in the comma-separated list on the right-hand corners of each plot, where ^‡^indicates *P* < 0.05.

Similar to what was observed in the untargeted lipidomics analysis (Figure 5A), our targeted LC-MS analysis (Supplementary Database 14) revealed an increased S1P/C16 Cer ratio in each of the subclones (Figure 7, *blue circles*). This occurred because basal S1P concentrations were *elevated* in the cancer groups relative to the healthy control and this increase was significant in pA, pC, and pD cells (*P* < 0.05) (Supplementary Figure 9A, *blue circles*). In addition, C16 Cer concentrations were significantly *depleted* in pB, pC and pD cells relative to the healthy control (*P* < 0.05) (Supplementary Figure 9B, *blue circles*).

Baseline C16 Cer levels were slightly, but significantly (*P* < 0.05) *higher* in pA cells compared to hTert; however, pA samples exhibited the highest basal S1P concentrations of the entire experiment, which may have helped balance signaling effects of the elevated C16 Cer levels in these cells relative to hT (Supplementary Figure 9A–9B, *blue circles*). Basal S1P expression in pB cells was not significantly higher than hTert; however, basal C16 Cer expression was *lowest* in pB cells compared to all the other groups, which may have helped balance the less elevated S1P levels. The pC and pD cells displayed the highest average basal S1P levels and significantly low basal C16 Cer levels, suggesting that these groups altered S1P/C16 Cer metabolism from both ends of the pathway (Figure 1) to favor S1P production and suppress C16 Cer levels. Overall, our LC-MS method confirmed that the cancer subgroups were phenotypically distinct from one another at the level of the global lipidome. In addition, all four PSN-1 strains modified intracellular S1P and/or C16 Cer metabolite levels to some extent relative to the healthy control.

### Extracellular lipid profiles of PDAC subclones mirrored the intracellular lipid profiles

If intracellular S1P is elevated, extracellular S1P levels may also be elevated [59]. The FBS in our media has high concentrations of many lipid species including S1P [60], therefore we compared the lipids extracted from growth medium samples that were exposed to cell cultures versus the fresh media as negative controls. The negative controls (blanks) were prepared by performing modified B&D extractions on fresh complete cell medium (RPMI-1640 with L-glutamine and high glucose, 10% FBS, 1x PBS) that was incubated in empty culture dishes (no cells) under the same conditions (37° C, 5% CO_2_) for the same amount of time as the cell-exposed samples (12 hours). There were 113 different lipid species (Supplementary Database 12) in the resulting list of annotations that met our statistical criteria (see Materials and Methods) and used for a global extracellular lipid profiling (Figure 4D1–D3). A variety of lipids types were represented at different levels across the five cell types including prenols (4%), glycerolipids (6%), sphingolipids (6%), polyketides (7%), fatty acids (10%), sterols (11%), and glycerophospholipids (56%) (Figure 4D1).

We used hierarchal heat map clustering and a PCA to make general comparisons between the extracellular lipid profiles of each group relative to the blank (Figure 4D2-D3). The heat map (Figure 4D2) and PCA (Figure 4D3) depicted three distinct clusters or subgroups within the dataset made up of the lipid profile(s) of (1) blanks, (2) hT, (3) pA, pB, and pC cells, and (3) pD cells. Above all, the heat map and PCA illustrated considerable degrees of variance between the global lipidome of the blank relative to samples exposed to cell culture (Figure 4D2-D3). We also observed that the inter- and extracellular lipidomes of all the cell groups were correlated. Similar to the intercellular lipid profiles (Figure 4C1, C3), pA, pB, and pC groups were more similar to each other while pD displayed the greatest degree of variance. This suggests that changes in nutrient lipid levels (Figure 2) can induce adaptive lipid metabolic reprogramming that greatly affect both intra- and extracellular lipidomes, as demonstrated by the pD cells (Figure 4D3).

This analysis provided a means to measure and compare *extracellular* S1P levels between cell types. Extracellular S1P levels from hT, pA, and pB cell cultures were slightly higher on average, but not significantly different than the S1P measured in the blank (Supplementary Figure 9C, *blue circles*). On the other hand, pC and pD cells displayed significantly higher extracellular S1P concentrations relative to the blank (*P* < 0.05) (Supplementary Figure 9C, *blue circles*). This suggests that, S1P produced within pC and pD cells was exported at higher levels relative to the other groups. This has been shown to promote pro-survival S1P signaling in an autocrine and/paracrine fashion [61]. These results provided further evidence that each PSN-1 subclone altered lipid utilization networks in unique ways to support pro-survival S1P signaling from within and/or outside of the cell.

### Pathway specific lipidomics and RNA-Seq analyses suggest a parallel shift in S1P/C16 Cer metabolism in PDAC subclones driven in part by Sphingosine Kinase 1

We identified several enzymes directly involved in perturbing the S1P/C16 Cer lipid ratio by RNA-Seq, including Acid Ceramidase (ACdase), Neutral Ceramidase (N-Cdase), Ceramide Synthase 5 (Cers5), S1P Phosphatases 1 and 2 (SPPases 1 and 2), S1P Lyase 1 (S1PL1), and Sphingosine Kinases 1 and 2 (Figure 5B). Among these enzymes, there were one or more nonsynonymous coding SNPs detected in ACdase, N-Cdase, CerS5, SK1, SK2, and S1PL1 (Supplementary Database 13). The median nscSNP densities of these five enzymes in each cell type (hT:0.043%, pA: 0.059%, pB:0.048%, pC: 0.068%, pD:0.068%) were similar to the median nscSNP densities of their corresponding genomes (hT: 0.059%, pA: 0.049%, pB:0.044%, pC: 0.043%, pD:0.039%). All four cancer groups displayed much higher SNP densities in ACdase (0.11%-0.15%), relative to ACdase enzymes in hTert (0.04%) (Figure 5C). *In silico* evaluations (see Materials and Methods) suggested two of these SNPs (C→T, G→T) (A→G, T→C, C→T) (Supplementary Database 13). However, there seemed to be no major effect on ACdase expression in the cancer groups relative to hTert, since ACdase mRNA levels were not significantly different from hT in any of the cancer groups (Supplementary Figure 10A).

One potentially significant polymorphism foreseen in this analysis was an L→P polymorphism repeatedly detected in position 237 of SK1 isoform 2 (SK1-2) [62] in pA cells (Supplementary Database 13). Residue 273 in SK1-2 is the equivalent of 187L in SK1-1, which is involved in an alpha helix in the C4 region of the C-terminal domain next to the sphingosine binding pocket [45]. The prediction tool suggested that this 273L→P polymorphism detected in the pA cells was probably damaging. This prediction is appropriate since switching from a more flexible leucine to an inflexible proline could break the helix, potentially affecting binding or substrate affinity to the proximal sphingosine binding site. While the SNP analysis provided some evidence that pA was biochemically reprogrammed in slightly different ways with respect to the pathway of interest, it did not provide any significant evidence of genetic forces driving the major shift S1P/C16 observed across the four cancer groups relative to hTert (Figure 5A).

We looked further into our RNA-Seq data to determine whether SK1 or any of the other sphingolipid metabolic enzymes were transcribed differently in the cancer groups relative to the healthy control. The normalized mRNA levels of the eight sphingosine metabolic enzymes were compared on a heat map (Figure 5D) and PCA plot (Figure 5E). According to the heat map, SPPase2 and SK1 mRNA levels appeared to be most altered relative to the other six enzymes (Figure 5D). Interestingly, these two enzymes catalyze opposite reactions in the C16 Cer/S1P metabolic pathway; SPPase2 dephosphorylates S1P to form sphingosine whereas SK1 phosphorylates sphingosine to form S1P (Figure 5B). Hence, we predicted that *Step 2* (Figure 5B), wherein sphingosine was either phosphorylated or dephosphorylated, was a critical point of control in the S1P/C16 Cer metabolic pathway with respect to driving differences between the healthy and cancer groups. The Dunnett's test was used to determine significant differences in mRNA levels of each enzyme between the cancer groups relative to the healthy control (Figure 5F–5G, Supplementary Figure 10). There were no clear trends in SPPase2 mRNA levels among the cancer groups or significant differences relative to the healthy control (Figure 5F). On the other hand, SK1 mRNA levels were *depleted* in all four cancer groups relative to hTert and this difference was significant (*P* < 0.05) in all but the pB cells (Figure 5G). This suggests that SK1 may be regulated differently in PSN-1 clones relative to healthy cells and perhaps may be linked with the shift from healthy to cancerous sphingolipid metabolism.

The PCA of [mRNA] depicted clear separations between hTert and PSN1, as well as subclones whose nutrients were changed (pB, pD) versus unchanged (pA, pC) (Figure 5E). We hypothesized that SK1 plays an important role in differentiating the “healthy” phenotypes exhibited by hTert from the cancer groups by playing a key role relative to other sphingolipid modifying enzymes in regulating S1P/C16 Cer metabolism in response to different metabolic stresses. We also anticipated that each cancer group may achieve a modified S1P/C16 Cer axis to promote clonal survival in different ways, especially since there were variations in sphingolipid enzyme expression between cancer groups subjected to different metabolic pressures (Figure 5, Supplementary Figure 10).

### SK1 activity may be modulated by a combination of increased total concentration and ERK2-mediated phospho-activation in pancreatic cancer subclones

**Figure 6:**
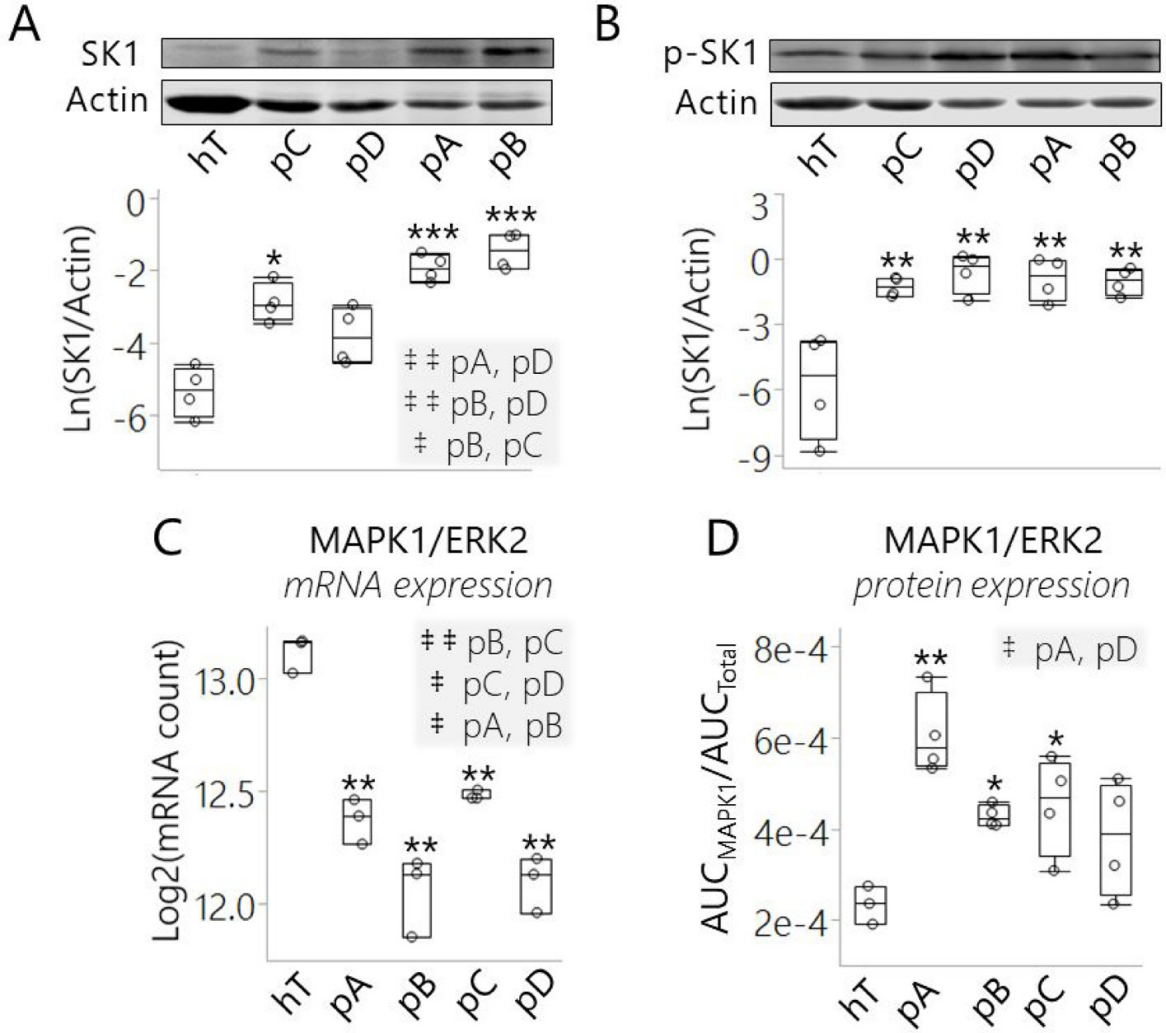
SK1 expression and/or ERK2-mediated phosphorylation was increased in pancreatic cancer subclones relative to healthy control cells (**A**, **B**) Representative Western blots and relative concentrations of (A) total SK1 and (B) phosphor-activated SK1 enzymes (p-SK1) in cancer subclones and hT cells lysates. Western blots were performed in biological quadruplet and actin was used for loading controls. Quantitation of each replicate is represented as (A) Ln(SK1/Actin) and (B) Ln(p-SK1/Actin) in the box plots below the respective representative Western blots. (A–B) The ^***^indicates *P* < 0.0001, ^**^indicates *P* < 0.001, and ^*^indicates *P* < 0.05 with the Dunnett's Test used to compare all cancer groups to the healthy control (hT). Tukey-Kramer Tests were used to determine significant differences between cancer groups; pairs that were significantly different are highlighted in the comma-separated lists on the right-hand corner of (**A**), where ^‡ ‡^indicates *P* < 0.005 and ^‡^indicates *P* < 0.05. (**C**) Box plot of normalized mRNA levels of MAPK1/ERK2 in cancer subcultures and hT cells measured by RNA-Seq. Measurements were collected in biological triplicate and represented on a Log2 scale. (**D**) Box plot of normalized MAPK1 protein concentrations measured by quantitative proteomics of cancer subcultures and hT whole cell lysates. Data are represented as the mean normalized MAPK1/ERK2 protein concentration (AUC_MAPK1_/AUC_Total_) of 3–4 biological replicates per group. (C–D) The Dunnett's Test was used to compare the cancer groups to the healthy control (hT), wherein ^**^indicates *P* ≤ 0.0003 and ^*^indicates *P* < 0.05. Tukey-Kramer Tests were used to determine which cancer groups differed from each other; pairs that were significantly different are highlighted in the comma-separated lists on the top right-hand corner of each plot, where ^‡ ‡^indicates *P* < 0.01 and ^‡^indicates *P* < 0.05.

Because SK1 was not detected in our global proteomics analysis, we used Western blotting to measure SK1 protein expression levels in each cell group (Figure 6A). SK1 concentrations varied greatly among the cancer groups, supporting our hypothesis that the different PSN-1 subclones may have used SK1 to dysregulate S1P/C16 Cer metabolism. Unlike SK1 mRNA, the average SK1 protein concentration was higher in all the cancer groups relative to hTert and this difference was significant in pA (*P* < 0.0001), pB (*P* < 0.0001), and pC (*P* < 0.05) (Figure 6A). These results suggest SK1 protein expression was increased to some degree to promote S1P synthesis in each PDAC subclone compared to the healthy control.

The results of our SK1 Western blots led us to question why SK1 *protein* expression (Figure 6A) seemed to be in the opposite direction of SK1 *mRNA* expression (Figure 5G) in the cancer groups relative to hTert. Indeed, we saw that SK1 *mRNA* levels were depleted while SK1 *protein* levels were increased. This may indicate that SK1 translation was post-transcriptionally elevated. In contrast to SK1, *both* mRNA and protein levels of Beta-actin (Supplementary Figure 11A1-A2) and Gamma-actin (Supplementary Figure 11B1-B2) in the four cancer groups were depleted relative to hTert. This suggests that, unlike SK1, cytoplasmic actin proteins were *transcriptionally* regulated in the PDAC cells. We also checked another cancer-promoting lipid modifying enzyme, fatty acid synthase (FASN), for which we had both RNA-Seq and protein quantitation data from our proteomics analysis. Both FASN mRNA and protein levels have been shown to be overexpressed in most human cancers including PDAC, making FASN an important disease biomarker [63]. Consistent with other research [20, 24, 63] both the mRNA and protein expression levels of FASN were significantly increased in PDAC cells relative to the healthy control (*P* < 0.01) (Supplementary Figure 12A, 12B). Based on these results, we proposed that SK1 protein expression, as opposed to other differentially expressed species in the cancer groups like actin and FASN, was post-transcriptionally elevated in the PDAC subclones.

To compare activation levels of SK1 enzyme in the five cell groups, we used a phospho-SK1 (Ser225) polyclonal antibody to detect endogenous SK1 phosphorylation (Figure 6B). All the cancer groups displayed a significant increase in the amount of phosphorylated SK1 (p-SK1) relative to hTert (*P* < 0.001). The pA and pB cells exhibited the highest SK1 levels as well as increased SK1 phospho-activation relative to hTert; the pC cells displayed a smaller but significant increase in SK1 expression and phosphorylation compared to hTert; in contrast, total SK1 protein expression was not significantly increased but the median p-SK1 concentration was highest in the pD cells relative to hTert (Figure 6A, 6B). This suggests the perturbed S1P/C16 Cer ratio observed across PDAC subclones (Figure 5A) was achieved in unique ways by modulating SK1 concentration and activation levels in each subclone. Overall, the results of our Western blots indicate that overactive SK1 may be required to maintain sphingolipid metabolic reprogramming and signaling in PDAC cells under different forms of metabolic stress.

The high p-SK1 levels we observed in the cancer groups may be due to increased expression of the SK1 activating kinase, MAPK1/ERK2 (Figure 6C, 6D). ERK1/2 are key components of the pro-proliferative Ras/MAPK signaling pathway that is hyperactivated in many human cancers [64]. Both ERK1 and ERK2 activate SK1 via phosphorylation at Ser225 [59]. Although very similar in structure, ERK2 has much higher activating efficiency for human SK1 than ERK1 [59]. ERK2 mRNA expression was significantly reduced in all four cancer groups at different degrees relative to hTert (*P* < 0.001) (Figure 6C). However, ERK2 protein expression was *elevated* in all the cancer groups relative to hTert and this increase was significant in pA (*P* < 0.001), pB (*P* < 0.05), and pC (*P* < 0.05) cells (Figure 6D). ERK2 was detected in all five cell groups in our RNA-Seq and proteomics analyses. These assays suggest that like SK1, ERK2 protein expression was post-transcriptionally increased in the cancer groups relative to hTert.

Increased p-SK1 levels (Figure 6B) may be attributed in part to this increase in the expression of its high-affinity activating kinase (ERK2) in PSN-1 cells relative to hTert (Figure 6D). Because protein synthesis is energetically costly, cancer cells under microenvironmental/metabolic stress can limit translation to a specific subset of mRNA's that code proteins best suited to support survival and disease progression [64]. Based on these results, we believe that SK1 and ERK2 were among this subset of preferred mRNA molecules to promote S1P synthesis and pro-survival signaling in the PDAC clones.

### SK inhibition normalized S1P/C16 Cer levels in distinct PDAC subclones

We developed a sphingolipid targeted LC-MS based assay to quantify the effects of SK1 inhibition on S1P/C16 Cer metabolism (Supplementary Database 14). Cells were treated with Sphingosine Kinase Inhibitor 2 (SKI-II, 4-[[4-(4-Chlorophenyl)-2-thiazolyl]amino]phenol) a non-lipid compound displaying selective, competitive inhibition of human SK1 and SK2 [65]. SK-II has also been shown to exhibit noncompetitive inhibition of ceramide dihydroceramide desaturase 1 (Des1), the final step in *de novo* synthesis of ceramide [66]. Average S1P concentrations were higher in the vehicle controls of all the cancer groups relative to hTert and this difference was significant in pA (*P* < 0.05), pC (*P* < 0.05), and pD (*P* < 0.05) cells (Supplementary Figure 9A). SKI-II treatment reduced average intracellular S1P levels in all four cancer groups relative to their corresponding vehicle controls and normalized intracellular S1P levels in all the cancer groups relative to the healthy control. On the other hand, SKI-II had virtually no effect on intracellular S1P concentrations in hTert cells. This suggests that SK1-mediated S1P production was initially higher in the cancer groups, but SKI-II effectively suppressed hyperactive levels of SK1 mediated S1P synthesis.

SKI-II treatment also reduced average *extracellular* S1P levels in all of the cancer subclones, though this decrease was only significant in pA cells (*P* < 0.05) (Supplementary Figure 9C). Moreover, SKI-II treatment normalized extracellular S1P levels in the two cancer groups whose baseline extracellular S1P concentrations were significantly higher than the blank (*P* < 0.05), i.e. pC and pD cells. Alternatively, SKI-II treatment led to an increase in intracellular C16 Cer levels in all of the cancer subclones relative to the corresponding vehicle controls, though this difference was only statistically significant in pA cells (*P* < 0.05) (Supplementary Figure 7B). This suggests that the decrease in SK1 driven S1P production from ceramide precursors allowed C16 Cer concentrations to increase in cells treated with SKI-II.

**Figure 7:**
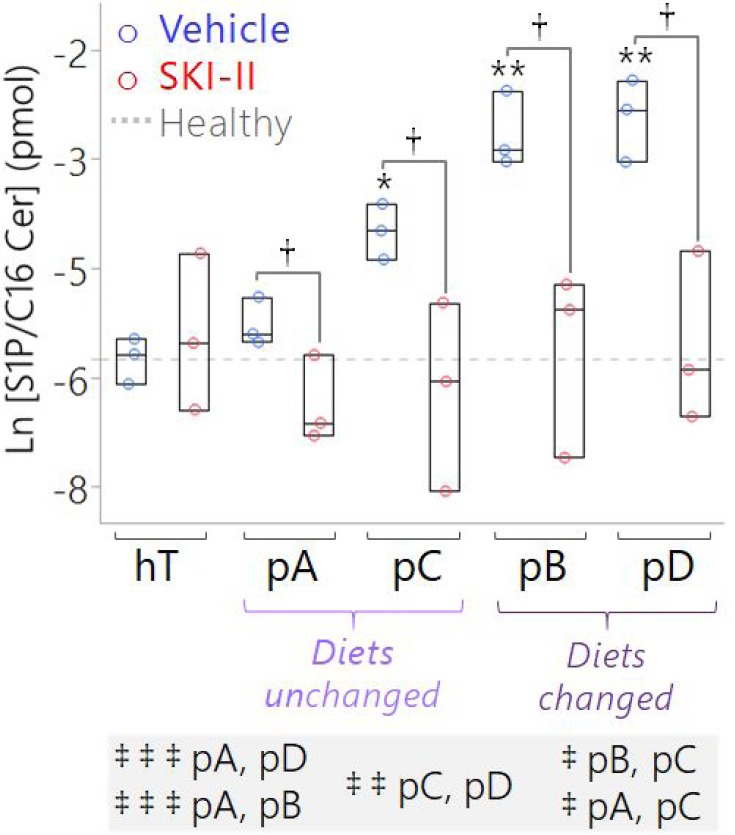
Targeted measurement of S1P/C16 Cer ratio suggests SK1 is a key driver of the conserved S1P:C16 Cer imbalance in pancreatic cancer subcultures, which may be corrected by SKI-II treatment Box plots of S1P relative to C16 Cer concentrations measured by LC-MS of lipids extracted from pancreatic cancer subclones and hT cell lysates treated with the vehicle (1x PBS) (blue circles) versus SKI-II (13 μM; red circles) for 12 hours. Both lipids were normalized to corresponding deuterated internal standards (S1P to 100 pmol of spiked S1P(d18:1-d7) and C16 Cer to 50 pmol of spiked Cer(d18:1-d7/16:0)). Data are represented as the ratio of the normalized S1P concentration (where pmol_S1P_/AUC_S1P_ = pmol_S1P-d7_/AUC_S1P-d7_) relative to the normalized C16 Cer concentration (where pmol_C16Cer_/AUC_C16Cer_ = pmol_C16Cer-d7_/AUC_C16Cer-d7_). The dotted gray line is the mean S1P/C16 Cer ratio of the hT Vehicle Control group, shown as a reference to represent a normal sphingolipid rheostat or the “healthy” balance between S1P and C16 Cer in non-cancerous human ductal pancreatic cells. The ^*^indicates *P* = 0.002 and ^**^indicates *P* < 0.0001 with the Dunnett's Test used to compare the Vehicle Control groups of the cancer subclones to the healthy (hT) Vehicle Control group. The ^†^indicates *P* < 0.05 with Student's t-tests comparing each Vehicle Control group to the corresponding SKI-II-treated group of the same cell type. The Tukey-Kramer test was used to compare all of the cancer groups to one another other; pairs of groups whose baseline (Vehicle Control) ratios of S1P/C16 Cer were significantly different from each other prior to SKI-II treatment are comma-separated in the highlighted list below the plot, where ^‡ ‡ ‡^indicates *P* ≤ 0.0002, ^‡ ‡^indicates *P* < 0.01, and ^‡^indicates *P* < 0.05.

The *ratio* of intracellular S1P/C16 Cer is considered a metric of the sphingolipid rheostat and serves as a critical biosensor for predicting cell fate and drug sensitivity [30, 41, 67–69]. On average, the ratio of S1P to C16 Cer was higher in all the cancer vehicle control groups relative to hTert and this increase was significant in pB (*P* < 0.00001), pC (*P* < 0.005), and pD (*P* < 0.00001) cells (Figure 7). This indicates that the sphingolipid rheostat was perturbed in all PSN-1 subclones, favoring S1P accumulation relative to C16 Cer. Note that this perturbation occurred at significantly different degrees between different subclones. Baseline S1P/C16 Cer in the pB and pD vehicle control groups were significantly higher than pA and pC vehicle controls (*P* < 0.01). This further supports our hypothesis that each subclone adapted different ways to maintain an imbalance in sphingolipid metabolism promoting pro-survival S1P levels relative to pro-apoptotic C16 Cer.

Despite the wide variations in basal C16 Cer and S1P levels, SKI-II significantly reduced the S1P/C16 Cer ratio in *all* the cancer subclones relative to the respective vehicle controls (*P* < 0.05) (Figure 7). Importantly, SKI-II treatment effectively normalized the average S1P/C16 Cer ratio in all groups whose basal S1P/C16 Cer ratios were significantly higher than the healthy control, including pB, pC, pD. Overall, these results together with the Western Blots (Figure 6) suggest that SK1 plays a significant role in regulating the perturbed sphingolipid rheostat in differentially evolved PDAC cells but may be corrected by SKI-II treatment. Next, we sought to determine whether elevated S1P/C16 Cer in PSN-1 clones contributed to their rapid growth rates and pro-survival signaling.

### SKI-II treatment reduced PDAC cell proliferation in a dose-dependent manner

**Figure 8:**
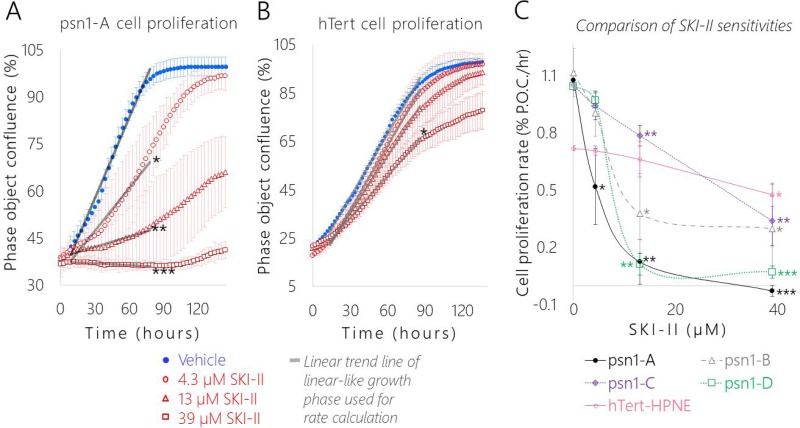
SK1 inhibition significantly slowed pancreatic cancer cell proliferation relative to the healthy control and each cancer subclones displayed a distinct level of dose-dependent SKI-II sensitivity Representative cell growth curves of (**A**) pA and (**B**) hT cells treated with Vehicle (1× PBS, blue dots) and the following concentrations of SKI-II: 4.3 μM (red circles), 13 μM (red triangles), and 39 μM (red squares). (A–B) Data are represented as the mean phase object confluence ± SEM of four biological replicates per group over time in hours. The gray lines are the linear trend lines of a portion of the linear-like growth period shown to illustrate how the proliferation rates for each cell group/condition were calculated. (**C**) Comparison of proliferation rates and sensitivity to increasing concentrations of SKI-II in pA (black dots), pB (gray triangles), pC (purple diamonds), pD (green squares), and hT (pink circles) cells. Growth rates were determined by calculating the slope of the linear-like growth phase of each group, where x = time (hours) and y = percent phase object confluence (% P.O.C.). Data are represented as the mean proliferation rate ± SEM of four biological replicates per group plotted against the SKI-II concentration in μM. (A–C) Student's *t*-tests were used to compare the proliferation rates of individual cell types treated with each concentration of SKI-II to the respective Vehicle Control group of the same cell type, where ^***^indicates *P* < 0.00001, ^**^indicates *P* < 0.005, and ^*^indicates *P* < 0.05.

We performed a live-cell confluence assay to generate growth curves of each cell type and measure the effects of SK1 activity on PDAC cell proliferation (Figure 8A, 8C; Supplementary Figure 13A-E). Cells were treated with a low (4.3 μM), medium (13 μM) or high (39 μM) dose of SKI-II and their growth rates were compared to corresponding vehicle controls. The vehicle control groups of all four PSN-1 subclones displayed rapid average basal proliferation rates, approximately 1.5 times faster than the healthy control (Figure 8C). The pB cells exhibited the highest average proliferation rate (1.12% confluence per hour), followed by pA (1.08% confluence per hour), pC (1.05% confluence per hour), pD (1.04% confluence per hour), and hTert (0.72% confluence per hour). SKI-II treatment significantly reduced the proliferation rates of all four cancer groups (*P* < 0.05) in a dose-dependent manner (Figure 8C). SKI-II also reduced average hTert growth in a dose-dependent manner, though the change in proliferation rate was only statistically significant at the highest dose of SKI-II (39 μM) in hT cells (Figure 8B, 8C, Supplementary Figure 13A).

Interestingly, each PSN-1 subclone displayed different levels of sensitivity to SKI-II treatment (Figure 8C). The pC cells seemed to be less sensitive to SKI-II, displaying the lowest average change in growth rate in response to the medium (13 μM) and high (39 μM) SKI-II doses compared to the other three subclones (Figure 8C). On the other hand, pA cells were significantly more sensitive to SKI-II treatment relative to the other subclones. Although the average proliferation rates of all four cancer groups dropped in response to the lowest dose of SKI-II (4.3 μM), this reduction was only statistically significant in the pA cells (*P* < 0.05) (Figure 8A, 8C). In addition, pA cells exhibited the greatest drop in proliferation in response to the high dose of SKI-II (39 μM) (*P* < 0.00001) relative to the other cell types (Figure 8C). This increased sensitivity may be due to the lower basal S1P/C16 Cer ratio displayed in the pA cells relative to the other cancer groups (Figure 7); a less extreme level of basal pro-proliferative S1P relative to C16 Cer may have made it so a lower concentration of SK1 inhibitor was sufficient to restore the healthy “hTert-like” balance in the sphingolipid rheostat in pA cells compared to the other cancer groups whose S1P/C16 Cer ratios were significantly higher (*P* < 0.05) (Figure 7). This suggests the cancer subclones adapted different levels of dependence on SK1 mediated S1P synthesis to support their rapid proliferation rates.

Treating with SKI-II was sufficient to either normalize or significantly lower the growth rates of all four PSN-1 clones relative to hT (Figure 8C). Indeed, just 4.3 μM SKI-II treatment was sufficient to normalize pA cell proliferation (0.52 ± 0.1% confluence per hour) to the rate of the hT vehicle control (0.72 ± 0.01% confluence per hour) (Figure 8C). The medium SKI-II dose (13 μM) was sufficient to significantly lower pB cell proliferation (0.38 ± 0.2% confluence per hour) and pD cell proliferation (0.11 ± 0.06% confluence per hour) relative to rate of the hT vehicle control (0.72 ± 0.01% confluence per hour) (Figure 8C). Even for the pC cells which seemed to be the most resistant to SKI-II treatment, the medium SKI-II dose (13 μM) was sufficient to nearly normalize pC cell growth (0.78 ± 0.05% confluence per hour) relative to the growth rate of hT cells treated with the low (4.3 μM) SKI-II dose (0.71 ± 0.02% confluence per hour) (Figure 8C). The average proliferation rates of all four cancer groups treated with the high SKI-II dose (39 μM) (pA: -0.03, pB: 0.30, pC: 0.34, pD: 0.13% confluence per hour) were also considerably lower than hT cells treated with the same dose (0.48% confluence per hour) (Figure 8C). These results suggest each differentially evolved PDAC subclone was dependent to some extent on SK1 enzyme activity to support their rapid basal proliferation rates.

### PDAC subclones displayed different levels of drug resistance, but SKI-II sensitized all subclones to mitochondria mediated apoptotic signals

**Figure 9:**
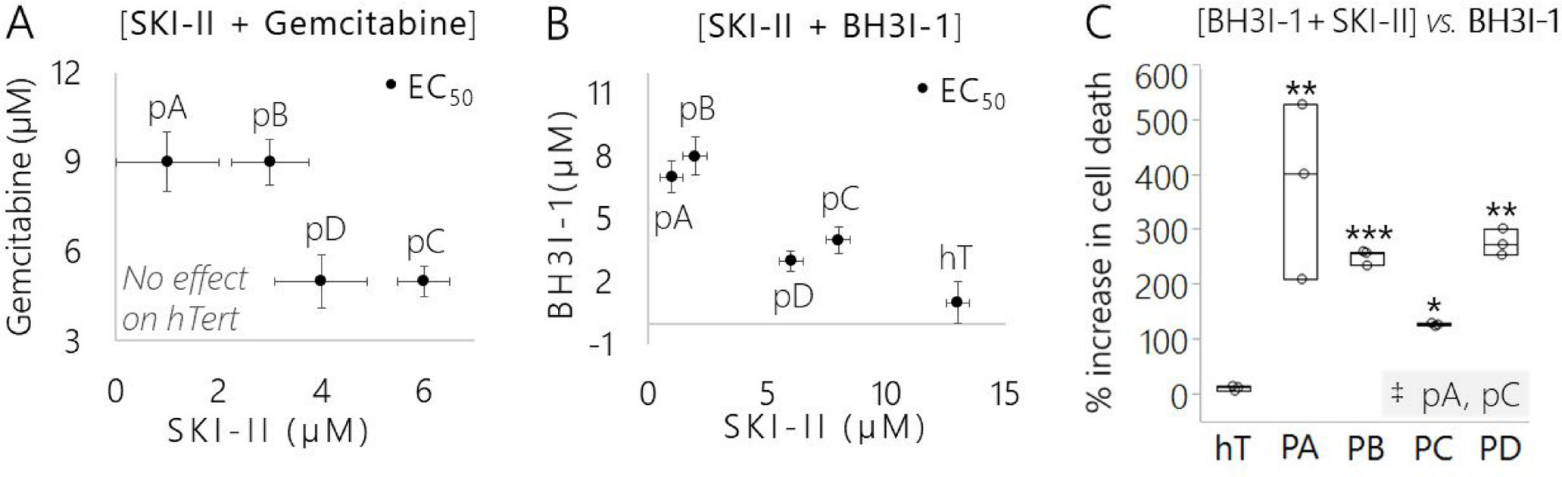
Pancreatic cancer subclones displayed different drug sensitivities yet SKI-II effectively sensitized each subclone to mitochondria-mediated apoptotic signals (**A**) Concentrations of control apoptosis-inducing treatment (SKI-II + Gemcitabine) that were required to achieve the EC_50_ of each cell group (with the exception of the slower growing hT cells where treatment was ineffective). Data are represented as the mean EC_50_ concentration of 3 biological replicates per group and error bars are drug tolerance (±) in μM. (**B**) Concentrations of targeted mitochondria-mediated apoptosis inducing treatment (SKI-II + BH3I-1) that were required to achieve the EC_50_ of each cell group. Data are represented as the mean EC_50_ concentration of 3 biological replicates per group and error bars are drug tolerance (±) in μM. (**C**) Comparison of the efficacy of SKI-II in enhancing cell death in response to mitochondria-mediated apoptotic signals across cancer subclones and healthy control. Data are represented as the percent increase in cell death of each group treated with SKI-II + BH3I-1 versus BH3I-1 alone (3 biological replicates per cell group/treatment). The Dunnett's test was used to compare between the percent increase in cell death of the cancer subclones to the healthy control (hT), wherein ^***^indicates *P* = 0.0004, ^**^indicates *P* < 0.008, and ^*^indicates *P* < 0.05. The Tukey-Kramer test was used to determine whether the percent increase in cell death of any of the cancer subcultures was different than any of the other cancer groups; the only pair found to be significantly different is highlighted in the bottom right-hand corner of the plot, where ^‡^indicates *P* = 0.011.

We performed a flow cytometric cell death assay with propidium iodide staining on PDAC subclones and hT cells treated with SKI-II, BH3I-1, or BH3I-1 combined with SKI-II (Figures 9; Supplementary Figure 14). BH3I-1 is a BH3 domain-only peptide activator of mitochondria-mediated apoptosis [70, 71]. Based on recent literature [72], we hypothesized that the SKI-II driven increase in C16 Cer levels (Supplementary Figure 9B) and decrease in S1P/C16 Cer (Figure 7) would enhance cancer subclone sensitivity to drugs, like BH3I-1, that directly induce mitochondrial outer membrane permeabilization via Bax/Bak. As a positive control, we compared BH3I-1 treated groups against cells treated with the nucleoside mimetic Gemcitabine (Gem). Unlike BH3I-1, Gem promotes apoptosis by inducing DNA damage [73]. Gem was selected as the control against BH3I-1 not only because it acts by a different mechanism to induce apoptosis in fast growing cells, but also because it is the most common chemotherapeutic used to treat PDAC with extremely low success rates [73].

We determined the half-maximal effective concentrations (EC_50_) of compounds in each cell group treated Gem, or BH3I-1 combined with SKI-II (Figure 9A, 9B). The EC_50_ of each subclone was distinct from the others for both combinatorial treatments. The pA and pB cells required a higher dose of Gem (9 μM) combined with a lower dose of SKI-II (1–3 μM) compared to pC (5 μM Gem + 6 μM SKI-II) and pD cells (5 μM Gem + 4 μM SKI-II) (Figure 9A). This suggests the pA and pB clones were more resistant to DNA damage-induced apoptosis compared to pD and pC clones and more sensitive to SK1 inhibition in the context of this treatment. Gem treatment was ineffective in hTert cells. This is likely due to the fact that hTert is a slower growing cell line (Figure 8C) and Gem targets fast-growing cells [74].

On the other hand, the BH3I-1 served as an effective apoptosis inducer in hTert cells. The hT cells required the highest dose of SKI-II (13 μM) combined with the lowest dose of BH3I-1 (1 μM) to achieve the EC_50_ (Figure 9B). Overall, much lower concentrations of SKI-II (1–8 μM) coupled with higher doses of BH3I-1 (3–8 μM) were required to achieve the EC_50_ in cancer subclones relative to hTert cells (Figure 9B). One interpretation of this could be that PDAC clones were resistant to mitochondria-mediated apoptosis, but more sensitive to SK1 inhibition compared to healthy cells. The pA and pB cells seemed to be the most resistant to BH3I-1, with an EC_50_ ranging from 7–8 μM and required the least amount of SKI-II (1–2 μM) compared to pC (4 μM BH3I-1 + 8 μM SKI-II) and pD (3 μM BH3I-1 + 6 μM SKI-II) (Figure 9B). This may mean pA cells were more resistant to mitochondria-mediated apoptosis compared to pC and pD, but more sensitive to SK1 inhibition in the context of this combination treatment. The pD cells required the least amount of BH3I-1 to achieve the EC_50_, suggesting that pD cells were more sensitive to mitochondrial apoptosis in the presence of SKI-II relative to the other cancer clones.

Using the doses determined for each cell line in our EC_50_ assay, we performed another set of cell-death experiments to specifically test whether SKI-II mediated SK1 inhibition enhanced sensitivity to BH3I-1 induced apoptosis. SKI-II induced a non-significant, but reproducible increase up to 10% in cell death across all of the cell groups in response to Gem treatment (Supplementary Figure 14A–14E). This may have been due to a counterproductive relationship between the mechanisms of action for Gem and SKI-II, since Gem targets fast growing cells and SK1 inhibition slows the proliferation rates of PSN-1 subclones (Figure 8C).

The percent of cell death in response to BH3I-1 alone was much lower compared to the response to Gem treatment alone in all cancer groups compared to hTert (Supplementary Figure 14A–14E). However, sensitivity to BH3I-1 was significantly increased in each cancer subclone treated with SKI-II relative to those treated with BH3I-1 alone (*P* ≤ 0.001) (Supplementary Figure 14B–14E, Figure 9C). The BH3 domain is a direct inducer of apoptosis via activation of pro-death Bcl-2 family members and does not rely on DNA damage checkpoint activation to kill the cell [71]. Therefore, the significant increases in cell death of PDAC cells treated with BH3I1 combined with SKI-II was likely driven by an increase in intracellular signaling of pro-apoptotic C16 Cer relative to S1P (Figure 7). Meanwhile, there was no significant change in cell death of healthy control cells treated with BH3I-1 versus hTert cells treated with the [BH3I-1 + SKI-II] combination treatment (Figure 9C; Supplementary Figure 14A). Indeed, the percent increase in cell death measured in all four cancer groups treated with the [BH3I-1 + SKI-II] combination versus BH3I-1 alone was significantly higher compared to the healthy control (*P* < 0.05) (Figure 9C).

There also were slight differences in the percent increases in cell death between each cancer subclone treated with [BH3I-1 + SKI-II] versus BH3I-1 alone (Figure 9C). Based on our Western blots and SK1 activity assay, this is likely due to variations in SK1 expression (Figure 6A) and activity (Figure 6B, Figure 7) between the different PSN-1 subclones and provides further evidence that each subclone adapted different methods to defend themselves against apoptotic signaling via SK1. Despite modifications in SK1 expression and regulation between the different subclones, the cell death assay reveals that SKI-II treatment effectively sensitized each subclone to mitochondria mediated apoptotic signals (Supplementary Figure 14B–14E). SK1 may serve as a key therapeutic drug target to ubiquitously enhance mitochondria mediated apoptosis in differentially reprogrammed PDAC subclones.

## DISCUSSION

To promote stress tolerance, isogenic clones derived from the same originating cancer cell can adopt distinct metabolic signatures leading to inter- and intra-tumor differences in disease progression, and drug resistance [3, 75, 76]. Targeting pro-cancer pathways that are selectively preserved throughout stochastic and environmentally induced metabolic reprogramming will improve treatment outcomes in aggressive and therapeutically unresponsive cancers like pancreatic ductal adenocarcinoma (PDAC) [3, 77]. This study was designed to investigate these preserved pro-cancer pathways in a PDAC line (PSN-1) under different forms of stress (Figure 2).

**Figure 10:**
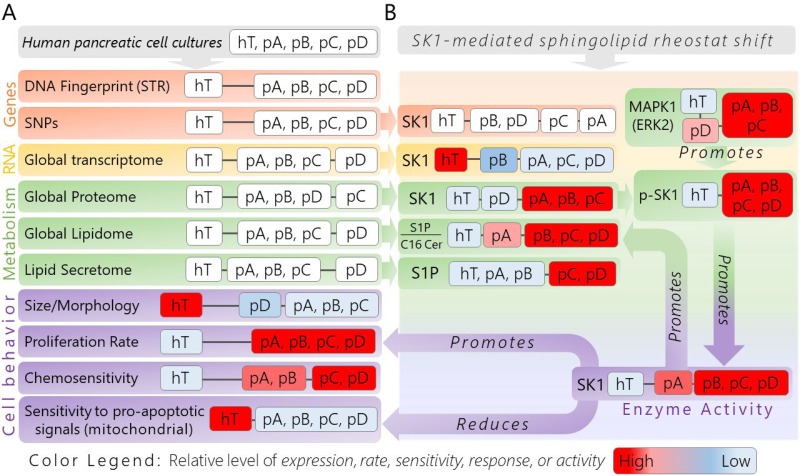
Divergence tree of genotypic and phenotypic analyses of isolated pancreatic cancer subcultures (pA, pB, pC, pD) and healthy control cells (hT) revealing nongenetic heterogeneity and a conserved, pro-cancer sphingolipid metabolic pathway mediated by SK1 (**A**) The experimental groups used to investigate pro-cancer adaptations in this study are shown in the gray box (top). This is followed by each parameter used to broadly compare genotypes and phenotypes of each cell group, as well as to determine at which level of expression common pro-cancer adaptations were present. The orange boxes, labeled “Genes”, represent assays performed to investigate genetic differences between each group. The yellow box, denoted “RNA”, represents our RNA-Seq analysis used to compare mRNA expression levels between groups. The green boxes, labeled “Metabolism”, represent assays to investigate metabolic features of each cell group, including protein and lipid (intra/extracellular) expression levels. The purple boxes, labeled “Cell behavior”, represent assays performed to measure major physical cancerous phenotypes/responses resulting from biochemical influences. (**B**) Boxes in this panel represent specific measurements of compounds that emerged from each global/omics assay related to the SK1-driven shift in S1P/C16 Cer metabolism observed to some degree in each cancer subclone. Relative to the healthy control, these biochemical influences promote SK1 activity in the cancer subclones in different manners in order to regulate cancerous behaviors, including proliferation and response to pro-mitochondria mediated apoptotic signals. (A–B) The distance between cell groups denoted by the black linker lines between each white box represent how closely the groups in each box were related with respect to the indicated assay. The color legend is shown to summarize general observations made in each assay, where appropriate; the boxes with groups showing high levels of expression, rate, sensitivity, response, or activity relative to the other cells are colored bright red; those cells showing very low levels of the respective assay metric relative to the other groups are colored light blue, while groups that were somewhere in between are colored with shades of red or blue toward the middle of the red→blue color spectrum.

Two PSN1 subclones (pA and pC) were maintained without changes in the nutrient formulations while two (pB and pD) received modified formulations to test for response to modified nutrient status (Figure 2). We found that there were detectable morphological changes after one month (approximately 10 passages) and subjected the subclones to a battery of assays. Each assay also compared these subclones against a ‘healthy’ control line for reference. The results of each assay are summarized in a divergence tree depicting commonalities and differences in global genotypic and phenotypic trends observed between groups (Figure 10A). Although there were no significant changes in DNA sequence between PSN-1 subclones, they exhibited multiple levels of phenotypic variation, including shifts in global mRNA, protein, and lipid expression levels as well as sensitivity to anti-cancer drugs.

The mRNA, protein, and lipidomics data also identified the S1P signaling pro-cancer metabolic/signaling pathway that may be mediated predominantly by the sphingolipid modifying enzyme Sphingosine Kinase (Figure 10B). Modified S1P/C16 Cer metabolism was conserved in different manners across the four differentially reprogrammed cancer subclones. Our proliferation and cell death assays further suggest that this pathway serves as an equally effective therapeutic target of SKI-II in each subclone by suppressing proliferation rates and enhancing mitochondria mediated apoptosis with no damaging effects on healthy control cells.

Our mRNA expression data suggest that differences in cancer subclone behavior were driven by changes in S1P metabolism (Figure 5D). Sphingosine Kinase 1 catalyzes the final step of S1P synthesis from ceramide precursors (Figure 1) and has previously been shown to play an important oncogenic role [46]. Surprisingly, the mRNA expression levels of SK1 were significantly reduced in all four cancer groups (Figure 5G). We used Western blots to measure the relative levels of total SK1 (Figure 6A) as well as active p-SK1 enzymes phosphorylated at Serine-225 (Figure 6B). SK1 protein concentrations varied between the four subclones and were significantly different between certain pairs, including pA/pD (*P* < 0.005), pB/pD (*P* < 0.005), and pB/pC (*P* < 0.05) groups (Figure 6A). All four cancer subclones displayed higher levels of SK1 protein expression levels relative to the healthy control, though this difference was only not significant in pD (Figure 6A). These data combined with the RNA-Seq (Figure 5G) suggest that SK1 protein expression was post-transcriptionally increased to promote S1P synthesis in the cancer groups relative to hT cells. SK1 mRNA levels may have been reduced due to continual translation/overuse to maintain high SK1 protein levels in the cancer cells relative to hTert. Elevated SK1 protein levels may trigger a negative feedback loop to suppress unnecessary SK1 mRNA production which could have also contributed to the reduced SK1 mRNA levels in the cancer cells compared to hTert.

Inactive SK1 is generally found in the cytosol away from its lipid substrates [59]. After activation through ERK2 mediated phosphorylation at Ser-225, SK1 relocates to the plasma membrane where it localizes onto functional lipid-raft subdomains for its catalytic activity [59]. Activating levels of SK1 (p-SK1) were significantly higher in all the cancer groups relative to the healthy control (*P* < 0.001) (Figure 6B). ERK2 protein expression was also higher in all the cancer groups relative to hTert and this difference was significant in pA (*P* < 0.001), pB (*P* < 0.05), and pC (*P* < 0.05) cells (Figure 6D). We thus propose that each cancer subclone achieved lower pro-apoptotic C16 Cer and higher pro-inflammatory S1P signaling (Figure 7, Supplementary Figure 9) through a combination of greater SK1 protein expression and/or increased SK1 activity levels through ERK2 mediated phosphorylation (Figure 6A, 6B).

**Figure 11:**
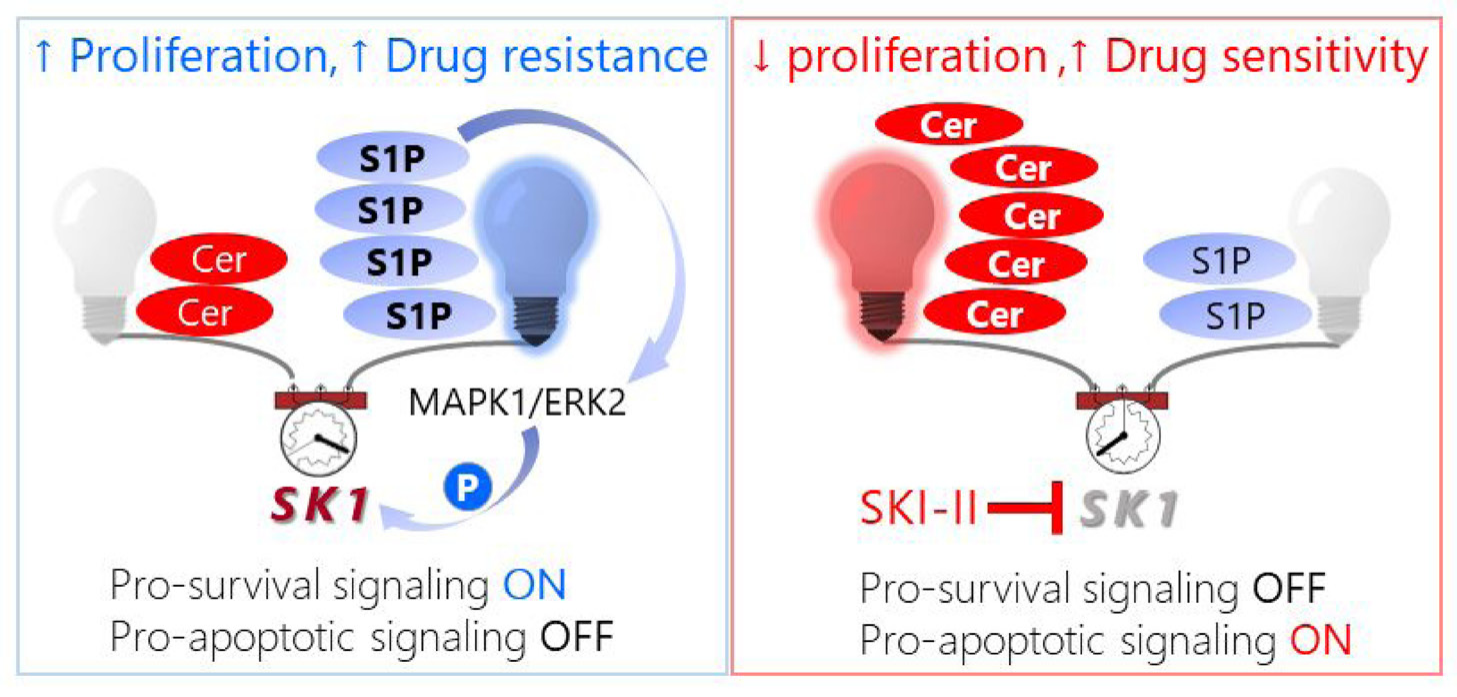
Schematic model of pro-survival S1P signaling in pancreatic cancer cells (left) followed by a shift in the sphingolipid rheostat toward C16 Cer-driven pro-apoptotic signaling induced by SKI-II treatment (right)

Multiple lipid extraction and mass spectrometry techniques confirmed that all four subclones maintained an increase in the ratio of intracellular S1P relative to C16 Cer, but at different levels (Figure 5A, Figure 7). This result was intriguing not only because C16 Cer and S1P are interconvertible metabolites (Figure 1), but also because they have been shown to exhibit competing bioactive capacities in cancer [30]. Together, C16 Cer and S1P seem to make up a critical rheostat between pro-survival versus pro-apoptotic signaling pathways in these differentially modified PDAC subclones (Figure 11). An increase in the level of pro-survival S1P molecules relative to pro-apoptotic C16 Cer has been shown to promote cancerous phenotypes like proliferation, stress tolerance, and resistance to ceramide-mediated apoptosis by activating intracellular targets including TRAF2, an essential E3 ubiquitin ligase in the pro-proliferative TNF-α/NF-κB signaling pathway [30]. S1P is also known to bind Prohibitin 2 (PHB2), a conserved protein responsible for mitochondrial membrane assembly and integrity [78], while C16-Cer may promote the mitochondria-mediated apoptotic pathway [35]. We considered the increased S1P/C16 Cer ratios in the cancer groups (Figure 7) representative of a cancer-promoting shift in the sphingolipid rheostat. We hypothesized that this shift in S1P/C16 Cer metabolism was used as an important stress tolerance mechanism of PSN-1 that was selectively conserved at various degrees in each isolated subclone.

To investigate this hypothesis, we tested the effects of pathway inhibition on S1P/C16 ratios and drug sensitivity. The subclones all responded to pathway inhibition (Figure 7), but with different EC_50_'s (Figure 9). In pA cells, we repeatedly detected a 273L→P polymorphism (Supplementary Database 13) found in the helix near the sphingosine binding site of SK1. This may have affected the Kd of SK1, leading to a reduction in the S1P/C16 Cer ratio in pA cells relative to the other cancers. On average, *extra*cellular S1P levels suggest S1P may be exported from pC and pD cells, which has been shown to promote autocrine/paracrine proliferative signaling [61]. Nonetheless, the highly variable S1P/C16 Cer levels observed in the other subclones did not seem to have a genetic origin (Figure 7, Supplementary Figure 9).

Overall, these initial implications from our targeted measurement of S1P/C16 Cer confirmed that although the sphingolipid rheostat was perturbed to some extent in each cancer subclone, increased levels of S1P may be used to hyperactivate both intra- and extracellular pro-proliferative targets. SKI-II treatment significantly reduced the S1P/C16 Cer ratio in all four cancer groups (*P* < 0.05), normalizing S1P/C16 Cer levels relative to the healthy control. Our Western blot data (Figure 6) suggests that SK1 concentration and phospho-activation is a major factor driving the S1P/C16 Cer imbalance in these cancer cells (Figure 7). Previous studies also report that SK1 is elevated in many human cancers and these elevated levels contribute to cancer development, drug resistance, and poor prognosis [38, 39, 42, 44, 66, 79–81]. While SKI-II is considered a selective inhibitor of SK1 [65], it also inhibits sphingosine kinase 2 and has been shown to have off-target effects on ceramide dihydroceramide desaturase 1 (Des1), leading to reduced S1P accumulation in gastric cancer cells [66]. So, SKI-II may inhibit S1P synthesis directly by blocking SK1 and SK2 or indirectly by slowing *de novo* synthesis of its metabolic precursor ceramide by simultaneously inhibiting Des1. This synergistic inhibition of the pathway may improve the effectiveness of SKI-II in normalizing S1P signaling across the different subclones (Figure 7).

Variations in S1P/C16 Cer levels (Figure 7) resulting from the SK1 mediated drive for increased proliferation may be linked to changes in cell size, shape, and production of membrane lipids to accommodate rapid growth rates of the cancer subclones (Figure 8C; Supplementary Figure 4C1; 3). While the cancer groups grew at nearly equivalent rapid rates, ranging from 1.04 to 1.12% confluence per hour (Figure 8C), there were modest but consistent variations in the general morphology and average sizes of individual subclones (Supplementary Figure 3A–3D). These morphological differences may be considered structural manifestations of the variations in mRNA and protein expression levels (Figure 4A, 4B), lipid metabolism (Figure 4C, 4D), and SK1 activity (Figures 6B, 7) induced by different internal and environmental stimuli that occurred during the subculturing process. For example, the slight increase in pD cell size may be linked to the shift in intra- and extracellular lipidomic profiles of pD cells relative to the other clones (Figure 4C, 4D). Lipid uptake has been shown to increase in some cancers [82]. The two-fold increase in serum lipids during the subculturing experiment (Figure 2) may have allowed pD cells to uptake and produce more lipids to accommodate larger plasma membranes. Indeed, pD cells exhibited the highest average concentration of cellular glycerolipids relative to the other cancer groups (Figure 4C1) which may have facilitated pD cell membrane growth (Supplementary Figure 1).

The generally smaller PDAC cell size (Supplementary Figure 3) may also be correlated with the reduced cytoplasmic actin mRNA and protein expression levels (Supplementary Figure 11A, 11B). The relative mRNA and protein concentrations of Beta-actin and Gamma-actin were depleted in all cancer groups relative to the healthy control (*P* < 0.01). Considerably higher actin mRNA and protein levels may be used to support the significantly larger cytoplasmic areas of hT cells relative to the cancers (Supplementary Figure 3F). In addition, pD cells were generally the largest of the four cancers and also exhibited the highest average actin concentrations relative to the other subclones (Supplementary Figure 11A2, S11B2). These data suggest that shifts in mRNA, protein, and lipid expression resulting from intrinsic and microenvironmental signals are intimately linked with hallmark cancer phenotypes like cell size and shape abnormalities.

Despite designed (Figure 2) and random variations in nutrient status (Supplementary Database 15), our study suggests that increased SK1 mediated S1P signaling may provide a pro-survival signaling environment to support cancerous behaviors in metabolically unstable PDAC subclones. To explore this concept further, we measured the effects of SK1 on PDAC cell proliferation. SKI-II treatment significantly reduced the growth rates of each cancer group in a unique dose-dependent manner relative to the healthy control (Figure 8; Supplementary Figure 13A–13E). These results suggest that enhanced SK1 activity was required to maintain rapid growth rates of all four PSN-1 subclones. Therefore, S1P signaling may serve as an important therapeutic target to uniformly suppress proliferation across pancreatic tumors made up of heterogeneous cell populations.

We tested whether SKI-II treatment was sufficient to enhance C16 Cer mediated pro-apoptotic signaling and drug sensitivity in the PDAC clones. One common mechanism by which ceramides communicate extracellular stress signals to the cell is by forming ordered, ceramide-enriched microdomains or lipid rafts [72]. Rafts on the outer layer can induce changes in the inner layer of the membrane, thereby transducing extracellular stress signals to pro-apoptotic effector molecules in the cytosol [30, 83]. In response to apoptotic stimuli, ceramides located in the outer mitochondrial membrane form ordered channels or pores, causing protein leakage from the intermembrane space and cytochrome c release, an initial step in the mitochondria-mediated apoptotic pathway [35].

Ceramide species have also been shown to regulate pro-apoptotic Bcl-2 family proteins and/or splice variants through multiple mechanisms [84, 85]. Without the second-messenger properties of ceramide, the ability of the cell to undergo programmed cell death becomes severely impaired or in some cases disabled entirely [38, 86]. Thus, active levels of ceramide are necessary to prevent tissue damage by minimizing the accumulation of damaged and oncogenic cells. Although the pathophysiological effects of ceramides in general have been reviewed thoroughly, the biological functions and techniques to measure *specific* ceramide species, such as C16 Cer, are less implicit [87], yet increasingly important for uncovering potential therapeutic applications [32].

Based on recent literature [72], we hypothesized that the increase in intracellular C16 Cer levels (Supplementary Figure 9B) induced by SKI-II may enhance PDAC cell sensitivity to mitochondria mediated apoptosis while suppressing S1P driven anti-apoptotic signaling. We performed a series of cell death assays to compare drug sensitives between each cell type and evaluate whether SK1 inhibition affected therapeutic responses to BH3I-1, a peptide activator of mitochondria mediated apoptosis relative to a standard chemotherapeutic, Gemcitabine (Figures 9, Supplementary Figure 14). Our results suggest the SK1 mediated shift in the S1P/C16 Cer ratio (Figure 7) provides some defense against mitochondria-mediated apoptosis in each cancer subclone. Much lower concentrations of SKI-II (1–8 μM) coupled with higher doses of BH3I-1 (3–8 μM) were required to achieve the EC_50_ in the PDAC relative to hTert cells (Figure 9B). The pA and pB cells seemed to be the most resistant to BH3I-1, with an EC_50_ ranging from 7–8 μM, and required the least amount of SKI-II (1–2 μM) in the combinatorial treatment compared to pC (4 μM BH3I-1 + 8 μM SKI-II) and pD cells (3 μM BH3I-1 + 6 μM SKI-II) (Figure 9B), suggesting that pA and pB cells were more resistant to mitochondria-mediated apoptosis compared to pC and pD. The pD cells required the least amount of BH3I-1 to achieve its EC_50_, suggesting they were more sensitive to apoptosis in the presence of SKI-II relative to other subclones (Figure 9B). Another way to interpret our EC_50_ results is to consider the individual concentrations of the two treatment components as less important compared to the total [SKI-II μM + BH3I-1 μM]. This may be the more definitive determinant of the EC_50_ since the two compounds are administered simultaneously and work toward the same general effect: an increase in apoptotic signaling. In this case, hTert required the highest combined sum total of SKI-II/BH3I-1 concentrations (14 μM), followed by pC (12 μM), pA (10 μM), pD (9 μM), and pA (8 μM) to achieve their individual EC_50_ levels (Figure 9B).

In the presence of SKI-II, there was a very modest increase in cell death resulting from Gemcitabine (Gem) treatment (Supplementary Figure 14). In order for a nucleoside analogue like Gem to have maximal effect, cells should be proliferating rapidly [88]. Since SKI-II treatment induced a potent anti-proliferative effect on the cancer clones (Figure 8C) it may have actively reduced Gem efficacy. On the other hand, BH3I-1 was a significantly more effective companion to SKI-II. SKI-II significantly enhanced the percent cell death of each cancer subclone exposed to BH3I-1 (*P* < 0.05) (Figure 9, Supplementary Figure 14), supporting the idea that increasing C16 Cer sensitized these cells to pro-apoptotic signaling at the mitochondria [38, 86].

Pancreatic cancer has historically been difficult to treat [89, 90] due to the resistant nature and the unique treatment-refractory environments established by individual tumors [91, 92]. Although Gemcitabine is the most common chemotherapeutic used to treat pancreatic cancer, the tumor response rate is just 12% [73]. Resistance to Gem presents major clinical challenges and new strategies to enhance PDAC drug sensitivity are in high demand [73]. In addition, inter- and intra-tumor heterogeneity resulting from differential cellular evolution reduces the predictability of individual treatment outcomes between isogenic experimental models and individual patients [9].

We showed that S1P signaling is a preserved pathway in metabolically reprogrammed PDAC cells and may be used as a ubiquitous drug target among isogenic subclones. Consistent with previous research [59], the model established in this study states that ERK2 initiates a pro-survival positive feedback loop by phosphorylating and activating SK1, thereby promoting S1P synthesis and suppressing relative C16 Cer concentrations. S1P in turn stimulates inter- and extracellular pro-inflammatory targets including the initial activating kinase ERK2 [59], leading to increased pancreatic cancer cell proliferation and drug resistance (Figure 11, *left panel*). SKI-II mediated SK1 inhibition increases pro-apoptotic C16 Cer levels relative to S1P, interrupting this S1P pro-inflammatory feedback loop, thereby reducing proliferation and minimizing anti-apoptotic defense systems (Figure 11, *right panel*). Concomitant suppression of S1P and enhancement of intracellular C16 Cer levels by inhibition of SK1 activity may serve as an effective strategy to restore a healthy balance between pro- and anti-apoptotic signaling in metabolically dynamic pancreatic cancers.

## MATERIALS AND METHODS

### Experimental design

The originating PSN-1 cell line and hTert cell line were obtained from Dr. David Bearss at Tolero Pharmaceuticals and were not tested for mycoplasma contamination after arrival. We developed a panel of differentially reprogrammed human ductal pancreatic cancer cells originating from the same genetic origin (PSN-1) [47] (Figure 2). The original PSN-1 cells were authenticated using the ATCC human cell line short tandem repeat (STR) profiling analysis [51] (Supplementary Figure 4B). These original cells were subcultured into four different randomly assigned groups: psn1-A (pA), psn1-B (pB), psn1-C (pC) and psn1-D (pD).

The first set of groups, pA and pC, were used to investigate stochastic, time-dependent factors influencing cancer evolution and were cultured separately in the baseline growth conditions in RPMI-1640 cell medium (Thermo Fisher cat # 11875093) with L-glutamine, high glucose, 10% FBS, and penicillin/streptomycin (PS) (1×) at 37° C and 5% CO_2_. The other groups, pB and pD, were cultured in new combinations of randomly assigned growth conditions during the same month to encourage environment-induced adaptions and metabolic reprogramming. The pB cells were maintained in DMEM cell medium with 10% FBS and 1× PS at 37° C and 5% CO_2_. The pD cells underwent the greatest nutrient change as they were cultured in IMDM cell culture medium with double the concentration of FBS (20%) and 1× PS at 37° C and 5% CO_2_. All three cell mediums used in this portion of the study (RPMI-1640, DMEM, IMDM) contained the same concentration of D-Glucose (25 mM). RPMI is generally the most different of the three in terms of contents and concentrations while DMEM and IMDM are more similar formulations (Supplementary Figure 2). The formulations differ the most in amino acid (Supplementary Figure 2A) and vitamin content (Supplementary Figure 2B). RPMI contains the greatest variety of amino acids; however, their concentrations tend to be lower than those in DMEM and IMDM. RPMI also contains the greatest variety of vitamins and is the only one of the three mediums to include Glutathione, an antioxidant that promotes cell growth and viability. After the month-long evolutionary period of culturing the cancer groups in these different cell culture mediums, frozen stocks of each group were prepared and stored in liquid nitrogen.

The growth conditions of each group were unified to the original growth medium (RPMI-1640 with L-glutamine and high glucose, 10% FBS, 1× PS) to obtain consistency of experimental conditions across all groups during the *in vitro* assays that followed. To minimize further cancer evolution/adaptive changes in each cell group from occurring throughout the study *after* the 1-month evolutionary period (Figure 2), cells from each group were passaged no more than 10 times before returning to an original frozen stock of the respective group.

### Cell size/morphology analysis

Light microscope imaging was used to compare cell shapes and sizes. ImageJ image analysis software was used to measure the cell areas of 40 different randomly selected cells on three different tissue culture dishes in each group (Supplementary Figure 3).

### STR profiling

Cell samples from each group were collected and STR profiles were generated using the ATCC Human Cell Line Authentication Service. Eight STR markers (plus amelogenin for gender determination) were amplified from cellular DNA via Polymerase Chain Reaction (PCR) and converted to the respective alleles by comparing to allelic ladders [51] (Supplementary Figure 4). These alleles were then converted to corresponding numeric values which were used to generate an STR profile of each group [51]. The STR Profile of each group was then compared to the corresponding reference profiles to determine the degree of relatedness (% match) to the original tissue [51].

### RNA-Seq analysis

The mRNA extracts were prepared from cell cultures of each group using the Direct-zol™ RNA MiniPrep Plus Kit. Samples were stored at −80° C for one week to one month. Three to four samples from each group with RQN values ≥8.0 and were selected for sequencing at the DNA Sequencing Center (DNASC) at Brigham Young University. There was a Poly-A enrichment prior to the library construction and libraries were sequenced using the HiSeq 250 Cycle Paired-End (125 cycles from each end) sequencing method.

Resulting sequencing data was downloaded and quality analyzed using the fastqc package [93]. All samples passed the major sequencing quality parameters. Reads were then aligned to the human genome (GRCh38) and assigned to features using an R script based on the Rsubread package [94].

### SNP analysis/SNP profiling and data analysis

Variant analysis of the RNA-seq data was conducted using custom R scripts based on the VariantTools Bioconductor package. Variants were first called individually for each sample and then results for all samples collated into a single table with presence or absence of a variant at every genomic position with a variant in at least one sample. A PCA analysis comparing samples was then conducted using logisticPCA, which is designed for binary datasets [95]. Nonsynonymous mutations were identified using the VariantAnnotation package [96] and their effects predicted using Polyphen2 [97].

### RNA expression profiling and data analysis

The *DESeq2* R/Bioconductor package was used to filter and normalize raw RNA-Seq data as well as to identify differentially expressed genes between groups [56]. The hierarchal heat map of the normalized RNA-Seq data was created using DESeq2 and the PCA plot was made using *ClustVis* [98].

### Protein expression profiling and data analysis

Proteomics samples were prepared from whole cell lysates collected from cell cultures of each group using an on-filter trypsin digest procedure. Cells were first grown to 70–80% confluence on 15cm tissue culture dishes. The cell medium was aspirated and cells were washed with 1x PBS. Cells were trypsinzed and pelleted by centrifugation for 5 minutes at 1200 rpm. The pellet was gently washed with 1× PBS. Pelleted cells were lysed and protein was denatured in 6 M guanidine/HCl 100 mM Tris/HCl (pH 8.5) on a tissue homogenizer for 30 seconds. Total protein was quantified using a bicinchoninic acid (BCA) assay. Fifty μg of protein from the sample was transferred to a 30 kD spin filter and washed 2–3 times with 6 M guanidine/HCl 100 mM Tris/HCl (pH 8.5). Disulfide bonds were reduced using dithiothreitol and alkylated using iodoacetamide. The filter was washed twice with 25 mM ammonium bicarbonate. Proteins were re-suspended in 25 mM ammonium bicarbonate (pH~8) and digested overnight using Pierce MS-Grade Trypsin. The trypsin digest was quenched and peptides were spun through 30kDa filters via centrifugation. Samples were dried in a vacuum evaporator and re-suspended in 50 μL of 3% acetonitrile/0.1% formic acid. Proteomics data was collected from each sample on a Lumos Orbitrap (Thermo) mass spectrometer. To reduce the influence of time-dependent fluctuation or cross contamination from run to run, the sample analysis schedule was randomized using a random number generator.

PEAKS Studio software was used for *de novo* sequencing to identify proteins in our raw MS data as well as to quantify, filter (quality-control) and normalize our label-free quantitation data for each protein [99]. Peptides were identified from MS/MS spectra by searching against the Swiss-Prot human database. Protein annotations with at least 2 unique peptides and a false discovery rate less than 1% were included in the comparative quantitative analysis. We used *t*-tests corrected for multiple comparisons using the Benjamini-Hochberg method to determine significant differences in protein concentrations between groups. The hierarchal heat map of the normalized protein concentrations was created using *DESeq2* [56] and the PCA plot was made using *ClustVis* [98]. We used t-tests to determine significant differences between protein concentrations in each group; *p*-values were corrected for multiple comparisons using the Benjamini-Hochberg procedure. Protein concentrations were considered significantly differentially expressed if both the *p*-value and corrected *p*-value were <0.05. The concentrations of differentially expressed proteins were compared between groups by calculating the fold change in concentration (mean concentration of each individual group relative to the mean concentration in the other groups). If the fold change was >1 the protein was considered upregulated and if the fold change was <1 the protein was considered downregulated.

DAVID Bioinformatics Functional Annotation Tools were used identify enriched functional-related gene groups in each list of significantly differentially expressed proteins [17]. The top three gene ontologies with the highest enrichment scores were considered relevant but only considered significantly enriched if the Benjamini score for enrichment calculated by the functional analysis tool was <0.05 [17].

### Shotgun lipidomics assay development and data analysis

Lipids with mass difference from LMSD <50 ppm were used in quantitative analysis

Several extraction methods were evaluated to determine which method would best sample each of the major lipid classes and introduce the least amount of bias into our mass spectrometry analysis. All methods had differential extraction efficiency with differences in the observed lipid categories and classes. One complication with mass spectrometry techniques is that it requires a charged molecule to make measurements. Many lipids do not have an intrinsic charge, but addition of ammonium acetate in the extraction protocol increased the coverage of lipids from all categories in both positive and negative ion modes. Based on the initial lipid classifications we determined that a modification to Bligh and Dyer extraction with addition of isopropanol and an ammonium acetate adduct [100] resulted in the most reproducible, broad coverage of the major lipid categories. Total unique lipid identifications that met our criteria were compared for each of the extraction methods. We repeatedly identified the largest number of lipid species using the modified Bligh and Dyer technique [100]. The sample preparation procedure used in this shotgun lipidomics assay is explained in the following paragraph.

Cell pellets were re-suspended in 1.5 mL cell lysis buffer (0.1M Tris-HCl at pH 7.6) and homogenized by circular cut tissue homogenizer (Omni) and vortex (30 seconds at 850 RPM). The homogenate was then transferred to glass vial (4.5 dram) where a two-phase extraction was completed to remove the lipid constituents [100]. Sample collection and homogenization steps were performed under cold collection environments and under nitrogen to reduce oxidation. The final extraction mixture contained the 1.5 mL aqueous homogenate and then an additional 3 mL of chloroform/methanol/isopropanol (3:1:1.25, v/v/v). A larger extraction batch with the Bligh and Dyer was extracted over 24 hours with shaking gave the closest match to a spiked standard. The organic lipid containing layer was then concentrated under reduced pressure at room temperature. The concentrated lipid extract was divided into two separate samples for comparison of adduct effects. The half sample analyzed without adduct was diluted with organic phase solution chloroform/methanol/isopropanol (3:1:1.25, v/v/v) with 0.1% formic acid (Thermo) at a 9-fold dilution by volume (9:1, solution: organic layer extraction) to a total volume of 250 μL immediately prior to data collection. The half sample analyzed with adduct was diluted with the same organic phase solution with 0.1% formic acid and ammonium acetate (1.5 mM) to promote ionization of some of the neutral lipid species. The sample (with and without adduct) was run in both positive and negative instrument modes to increase specificity and variety of lipids through intra-source selection of differentially ionizable species.

To reduce the influence of time-dependent fluctuation or cross contamination from run to run, the sample analysis schedule was randomized using a random number generator. In this way if the wash cycle between sample runs did not remove all the contaminants from the capillary line, the contaminated peak should not appear more than once in the technical replicates and is thereby removed by the data analysis filters. Sample (250 μL) was infused at 10 μL/min onto a Thermo LTQ-Orbitrap XL mass spectrometer using an IonMax ESI soft-ionization direct inject technique. During the infusion a high resolution (≥100,000) MS1 survey scan cycled through m/z “windows” (75–250 m/z, 250–400 m/z, 400–600 m/z, 600–800 m/z, and 800–1800 m/z). The top 5 most intense ions from each scan were selected for fragmentation, after an ion had been selected for fragmentation twice it was excluded from further MS/MS selection.

The typical data acquisition measured approximately 2000 different ions. Initial lipid identifications were assigned for each ion based on the parent mass of ion in the primary survey scan. To increase accuracy, the m/z of each ion was corrected according to the standard curve of the internal standards. The instrumental noise was determined as the baseline detection level across all spectra. Only those peaks that are estimated to be at least twice the level of the instrumental noise are included. The data files were analyzed using in-house developed module for the MSPIRE proteomics package [101] which compared the masses and fragmentation patterns against expected masses and fragments for the lipids within the LIPID MAPS database [102]. Ions which differed from the theoretical mass provided by the database by more than 50 ppm were removed from the data set. For quantitation comparisons across groups, MS intensity data for each lipid was normalized to the sum of all the species quantified in each sample. The normalized summed spectral intensity for each sample was considered a single quantitative data point each lipid.

### Intra/extracellular lipidomics LC-MS/MS analysis

We established a sphingolipid focused extraction technique and reverse-phase (RP) LC-MS/MS method based on the procedure developed by Bode *et al.* [57]. Cell cultures were plated onto 15-cm cell culture dishes in complete RPMI 1640 cell culture medium and incubated at 37° C, 5% CO_2_. Once cells reached 65–75% confluence, the cell medium was aspirated and cells were washed twice with 1xPBS. Medium was replaced with fresh, pre-warmed (37° C) complete RPMI 1640 containing 13 μM of SKI-II inhibitor or an equivalent volume of 1x PBS for vehicle controls. Cell cultures were incubated for 12 hours at 37° C, 5% CO_2_. After the incubation period, the medium was aspirated from the cells and transferred to a glass pear-shaped flask. Total lipids were extracted from the flask using the modified B&D technique [100], vacuum dried, dissolved in 100 μL of 4:1 (v/v) MeOH/CHCl_3_ and sealed under argon in glass MS vials. Meanwhile, cells on the plate were washed with 1x PBS two times, trypsinized, and pelleted via centrifugation at 1200 rpm for 5 min. The supernatant was decanted and the sphingolipid modified lipid extraction technique based on the B&G method [57] was performed on wet ice, in glass centrifuge tubes under argon gas to minimize lipid oxidation (procedure detailed below).

Cells were lysed via vortex in 1 mL of NaCl for 20 seconds. One mL of MeOH and 200 μL of 6 M HCl were added. The lysate was vortexed for 10 seconds. The organic phase lipid extraction was performed by adding 2 mL of CHCl_3_ to the sample which were vortexed for 2 minutes and then centrifuged for 3 minutes at 1900 g. The lower organic phase was transferred to a glass test tube. The phase extraction steps were repeated on the remaining aqueous layer in the sample and the resulting organic phase was combined with the first. CHCl_3_ was evaporated from the sample in a vacuum concentrator. The vacuum-dried lipids were dissolved in 100 μL of 4:1 (v/v) MeOH/CHCl_3_ and sealed under argon in glass MS vials.

Samples were analyzed via RP-LC-MS on a stepwise gradient using a Luna Omega 1.6uM Polar C18 100Å LC Column, 150*2.1mm (Phenomenex Part # 00F-4748-AN). The mobile phases were 1% Formic Acid (Buffer A) and 100% Methanol (Buffer B) run on the following gradient at 100 μL/min: 10%→100% Buffer B (0–5 minutes), 100% Buffer B (5–25 minutes), 100%→10% Buffer B (25–27 minutes) with a stop time of 45 minutes. Liquid chromatography was followed by positive ESI on a Dual Jetstream ESI source, MS/MS fragmentation using variable collision energy based on ion mass, and mass detection using an Agilent quadrupole-time-of-flight (QTOF) mass spectrometer. To reduce the influence of time-dependent fluctuation or cross contamination from run to run, the sample analysis schedule was randomized using a random number generator. To reduce sample carryover on the column, a blank containing 4:1 (v/v) MeOH/CHCl_3_ was run in between each sample. The injection needle was also washed twice with 48% acetonitrile/48% H_2_O/1% formic acid/1% cyclohexane followed by 99% isopropyl alcohol/1% cyclohexane to reduce sample carryover on the needle between each run.

We set up a workflow in the Agilent MassHunter Qualitative Analysis workstation to annotate signals in our raw MS data using the Metlin Lipids MS Database [103]. To verify these annotations, we measured the retention time (RT) alignment of each lipid by calculating the coefficient of variation (CV) of the respective RT across all the samples run on this method (6 per cell group = 30 samples total). We used the 500 lipids with the *lowest* CV of RT (<25%) in our global quantitative lipidomics analysis. Only 112 total lipids among the lipids identified in the cell medium samples met these criteria (CV of RT ≤25%) and were used for global extracellular lipid profiling.

### Western blot analysis

Cell pellets were lysed with ice-cold RIPA lysis buffer supplemented with protease inhibitor (Thermo scientific #A32965) and Phosphatase inhibitor (Thermo scientific #A32957). Protein concentrations of the clarified lysates were determined with the DC Protein Assay (BioRad). 50ug of total protein from each sample were resolved on 10% SDS-PAGE. Gels were then transferred to nitrocellulose membrane and were immunoblotted for proteins of interest (SK1 and p-SK1). Actin was used for loading controls. The following antibodies were used: Actin (C-2) *(Santa Cruz Biotechnology sc-8432)*, Anti-SPHK1 antibody (*Abcam ab71700)*, and SPHK1-Phospho-Ser225 Antibody (*Proteintech 19561–1-AP).* Proteins of interest were visualized and quantified by the Li-Cor Odyssey Classic or CLx imaging system and the Image Studio software package.

### Targeted S1P/C16 cer quantitative analysis

Cell cultures were plated onto 15-cm cell culture dishes in complete RPMI 1640 Cell Medium and incubated at 37° C, 5% CO_2_. Once cells reached 65–75% confluence, the cell medium was aspirated and cells were washed twice with 1xPBS. Medium was replaced with fresh, pre-warmed (37° C) complete RPMI 1640 medium containing 13 μM of SKI-II inhibitor or an equivalent volume of 1× PBS for vehicle controls. All cells were treated with an equal dose of the inhibitor to maintain sample uniformity, which was the highest concentration of SKI-II used in our EC_50_ estimation assay. We considered this dose representative of the concentration of SKI-II required to sensitize cells to a drug-induced effect on cell viability in a population of healthy pancreatic cells. Cell cultures treated with SKI-II and vehicle controls were incubated for 12 hours at 37° C, 5% CO_2_. After the incubation period, total lipids were extracted from the cells and cell medium followed by RP-LC-MS analysis using positive electrospray ionization using the methods described above (Intra/extracellular Lipidomics LC-MS Analysis). To increase the detection and accuracy of our S1P and C16 Cer measurements, we spiked deuterated and/or odd-chain internal standards into each sample immediately following the cell lysis step and switched to a targeted version of our RP-LC-MS method designed to specifically select protonated S1P and C16 Cer ions for MS/MS fragmentation. Diluted stock solutions of the internal standards were made by diluting in MeOH and the following volumes were spiked into each sample prior to lipid extractions: 20 μL of 2.5 μM C16 Cer-d7 diluted in MeOH, 20 μL of 2.5 μM C17 Cer diluted in MeOH, and 20 μL of 5 μM S1P in MeOH.

Our sphingolipid optimized sample preparation and LC-MS method significantly improved the singal:noise ratio of C16 Cer ions in all of our samples, greatly increasing the confidence of our annotation and quantitation of this particular target. Yet, we were concerned that the QTOF lacked sensitivity required to detect low S1P levels because it was not observed all our cell samples. In addition, the upper pressure limit on the pumps leading to our QTOF instrument limited our ability to run at pressures high enough to potentially increase the S1P signal:noise ratio. To further improve the consistency and accuracy of our S1P detection and quantitation, we reran our samples using an S1P targeted method with higher pressure pumps and a more sensitive triple-quadrupole (qQq) mass spectrometer at the Metabolomics Core Facility at the University of Utah. This method significantly improved the chromatography, signal intensity, fragment verification, and overall consistency of our S1P measurements in all our samples (Supplementary Figures 6B; 8A, 8B).

The identities of our two sphingolipid targets, C16 Cer and S1P, were confirmed by retention time alignment (Supplementary Figure 6A–6B) and MS/MS fragment verification (Supplementary Figures 7–8) with the corresponding internal standards. Quantitation of S1P and C16 Cer was performed by normalizing to the AUC of the corresponding internal standards initially spiked into the cell lysates (50 pmol of C16 Cer-d7, 50 pmol of C17 Cer, and 100 pmol of S1P-d7). The following equation was used for C16 Cer quantitation: (50 pmol)/(AUC_C16Cer-d7 or C17 Cer_) = (*x* pmol) / (AUC_D7C16Cer_), where *x* = [C16 Cer] (Supplementary Figure 6A). Note that C16 Cer was normalized to whichever internal standard had higher a signal:noise ratio in the MS run (C16 Cer-d7 or C17 Cer). The following equation was used for S1P quantitation: (100 pmol)/(AUC_D7S1P_) = (*x* pmol)/(AUC_S1P_), where *x* = [S1P] (Supplementary Figure 6B).

### Cell proliferation assay

Cells from each group were plated evenly on a 24-well tissue culture dishes and incubated overnight at 37° C, 5% CO_2_. Each dose of SKI-II inhibitor (*Santa Cruz Biotechnology cas 312636-16-1*) was prepared by serial dilution in complete RPMI 1640 Cell Medium (Thermo Fisher cat # 11875093). The SKI-II treated volumes of cell medium were sterilized on 0.2 μm filters and heated to 37° C. Wells containing adhered cells in the tissue culture dishes were aspirated and washed twice with 1x PBS. One mL of SKI-II treated medium was added to each well. Real-time phase object confluence was monitored over time using an Incucyte ZOOM^®^ Live-Cell Analysis System at 37° C, 5% CO_2_ and quantitative data were analyzed using the Incucyte ZOOM^®^ data analysis software to generate cell growth curves. Proliferation rates were determined by calculating the mean slope of the linear-like growth phases of 3–4 biological replicates per group.

### Flow cytometry cell death assays

Cell death assays were completed by plating cells in T-75 flasks (~10,000 cells/cm^2^) in log-phase growth and allowing a minimum of 6 hours for growth and adhesion before drug treatment. Growth media was replaced with fresh media prior to injection of drugs into culture flasks or plates. Cells were then treated for 12–24 hours with drugs. At the end of drug treatment, dead cells were removed and collected in a new plate. Cells were washed with 1× PBS and this was collected and combined into the new plate. Living adhered cells were removed from growth plates using 0.05% pH balanced trypsin at 37° C and then transferred to the new plate. Under dark conditions on ice a 1 mg/100 mL solution of propidium iodide (PI) was mixed with light shaking into the cell mixture and allowed 15 minutes to stain. PI is a DNA-binding fluorescent dye used to distinguish between live cells with intact membranes versus dead cells whose membranes are permeable to the dye [104]. Cell counts were then collected on a red/blue acoustically focused Applied Biosciences Attune flow cytometer at a scan rate of 200 μL/min using BL2-PI blue laser and BL1 blue laser. Data was analyzed with the Attune software.

### Quantitation and statistical tests

If not otherwise specified, figure development as well as data quantitation and statistical tests were conducted in Excel (dot plots, bar graphs, *t*-tests, Benjamini-Hochberg procedure) and JMP (box plots, *t*-tests, Dunnett's tests, Tukey-Kramer tests).

## SUPPLEMENTARY MATERIALS FIGURES

































## Abbreviations

hTert-HPNE: (hTert or hT)
psn1-A or pA: PSN-1 subclone group A
psn1-B or pB: PSN-1 subclone group B
psn1-C or pC: PSN-1 subclone group C
psn1-D or pD: PSN-1 subclone group D
SK1: Sphingosine Kinase 1
C16 Cer: Ceramide(d18:1/16:0), LipidMaps ID# LMSP02010004
C16 Cer-d7: Ceramide(d18:1-d7/16:0), LipidMaps ID# LMSP01050001
C17 Cer: Ceramide(d18:1/17:0)
S1P: Sphingosine-1-phosphate (d18:1)
S1P-D7: Sphingosine-1-phosphate (d18-d7:1)
SKI-II: Sphingosine Kinase Inhibitor 2
Gem: Gemcitabine
