# Supplementary material for: Imbalanced sphingolipid signaling is maintained as a core proponent of a cancerous phenotype in spite of metabolic pressure and epigenetic drift
Source: Oncotarget. 2019 Jan 11;10(4):449–79. doi: 10.18632/oncotarget.26533 (PMC6355186; doi:10.18632/oncotarget.26533)
Supplement: Supplementary file 1 [file oncotarget-10-449-s001.pdf]

Imbalanced sphingolipid signaling is maintained as a core proponent of a cancerous phenotype in spite of metabolic pressure and epigenetic drift

SUPPLEMENTARY MATERIALS

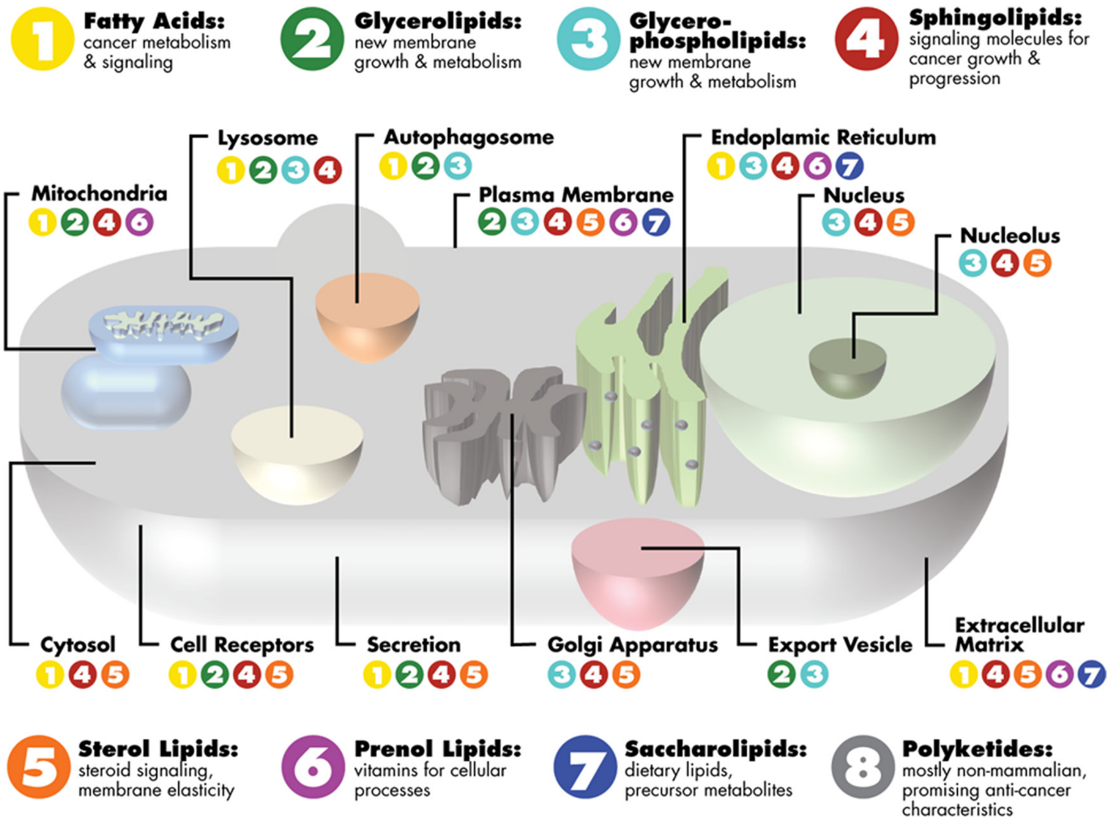

Supplementary Figure 1: General localization of lipids and cancer-promoting roles in the cell. At the top and bottom of this figure each lipid category/family is given a corresponding number (1–8) and brief description concerning their general involvement in supporting cancerous phenotypes. The major subcellular components have been labeled and the lipid families known to be found at each subcellular component have been listed through their corresponding numbers. The location and function of individual lipid species may change as the cell responds and adapts to stress.

A

Amino acid (mM)	RPMI (pA, pC)	DMEM (pB)	IMDM (pD)	RPMI vs. DMEM (mM)	RPMI vs. IMDM (mM)	DMEM vs. IMDM (mM)
Glycine	0.133	0.400	0.400	-0.267	-0.267	0
L-Alanine	0	0	0.281	0	-0.281	-0.281
L-Arginine hydrochloride	0	0.398	0.398	-0.398	-0.398	0
L-Arginine	1.149	0	0	1.149	1.149	0
L-Asparagine (freebase)	0	0	0.189	0	-0.189	-0.189
L-Asparagine	0.379	0	0	0.379	0.379	0
L-Aspartic Acid	0.150	0	0.226	0.150	-0.075	-0.226
L-Cystine 2HCl	0.208	0.201	0.292	0.006	-0.084	-0.091
L-Glutamic Acid	0.136	0	0.510	0.136	-0.374	-0.510
L-Glutamine	2.055	4.000	4.000	-1.945	-1.945	0
L-Histidine hydrochloride-H2O	0	0.200	0.200	-0.200	-0.200	0
L-Histidine	0.097	0	0	0.097	0.097	0
L-Hydroxyproline	0.153	0	0	0.153	0.153	0
L-Isoleucine	0.382	0.802	0.802	-0.420	-0.420	0
L-Leucine	0.382	0.802	0.802	-0.420	-0.420	0
L-Lysine hydrochloride	0.219	0.798	0.798	-0.579	-0.579	0
L-Methionine	0.101	0.201	0.201	-0.101	-0.101	0
L-Phenylalanine	0.091	0.400	0.400	-0.309	-0.309	0
L-Proline	0.174	0	0.348	0.174	-0.174	-0.348
L-Serine	0.286	0.400	0.400	-0.114	-0.114	0
L-Threonine	0.168	0.798	0.798	-0.630	-0.630	0
L-Tryptophan	0.025	0.078	0.078	-0.054	-0.054	0
L-Tyrosine disodium salt dihydrate	0.111	0.398	0	-0.287	0.111	0.398
L-Tyrosine disodium salt	0	0	0.462	0	-0.462	-0.462
L-Valine	0.171	0.803	0.803	-0.632	-0.632	0

B

Vitamin (mM)	RPMI (pA, pC)	DMEM (pB)	IMDM (pD)	RPMI vs. DMEM (mM)	RPMI vs. IMDM (mM)	DMEM vs. IMDM (mM)
Biotin	8.20×10^{-4}	0	5.33×10^{-5}	8.20×10^{-4}	7.66×10^{-4}	-5.33×10^{-5}
Choline Chloride	0.021	0.029	0.029	-0.007	-0.007	0
D-Calcium pantothenate	0.001	0.008	0.008	-0.008	-0.008	0
Folic Acid	0.002	0.009	0.009	-0.007	-0.007	0
Niacinamide	0.008	0.033	0.033	-0.025	-0.025	0
Para-Aminobenzoic Acid	0.007	0	0	0.007	0.007	0
Pyridoxine hydrochloride	0.005	0.019	0.020	-0.015	-0.015	-1.90×10^{-4}
Riboflavin	5.32×10^{-4}	0.001	0.001	-0.001	-0.001	0
Thiamine hydrochloride	0.003	0.012	0.012	-0.009	-0.009	0
Vitamin B12	3.69×10^{-6}	0	9.59×10^{-6}	3.69×10^{-6}	-5.90×10^{-6}	-9.59×10^{-6}
i-Inositol	0.194	0.040	0.040	0.154	0.154	0

C

Salt or other component (mM)	RPMI (pA, pC)	DMEM (pB)	IMDM (pD)	RPMI vs. DMEM (mM)	RPMI vs. IMDM (mM)	DMEM vs. IMDM (mM)
Calcium Chloride	0	1.802	1.486	-1.802	-1.486	0.315
Calcium nitrate	0.424	0	0	0.424	0.424	0
Ferric Nitrate	0	2.48×10^{-4}	0	-2.48×10^{-4}	0	2.48×10^{-4}
Magnesium Sulfate	0.407	0.814	0.814	-0.407	-0.407	0
Potassium Chloride	5.333	5.333	4.400	0	0.933	0.933
Potassium Nitrate	0	0	7.52×10^{-4}	0	-0.001	-0.001
Sodium Bicarbonate	17.857	44.048	36.000	-26.190	-18.143	8.048
Sodium Chloride	103.448	110.345	77.672	-6.897	25.776	32.672
Sodium Phosphate monobasic	0	0.906	0.906	-0.906	-0.906	0
Sodium Phosphate dibasic anhydrous	5.634	0	0	5.634	5.634	0
Sodium Selenite	0	0	9.83×10^{-5}	0	-9.83×10^{-5}	-9.83×10^{-5}
D-Glucose (Dextrose)	25.000	25.000	25.000	0	0	0
Glutathione (reduced)	0.003	0	0	0.003	0.003	0
HEPES	10.013	0	25.034	10.013	-15.021	-25.034
Phenol Red	0.013	0.040	0.040	-0.027	-0.027	0
Sodium Pyruvate	1.000	0	1.000	1.000	0	-1.000

Supplementary Figure 2: Comparison of components in cell growth mediums used to feed pancreatic cancer groups (pA, pB, pC, pD) during subculturing experiment. Concentrations of (**A**) amino acids, (**B**) vitamins and (**C**) salts and other components in RPMI 1640, DMEM and IMDM cell culture mediums and differences in the concentrations between each component in the three different formulations. (A–C) Cells in the table shaded red indicate differences between formulations while those shaded green indicate the concentrations of the component in the respective row are the same among the formulations.

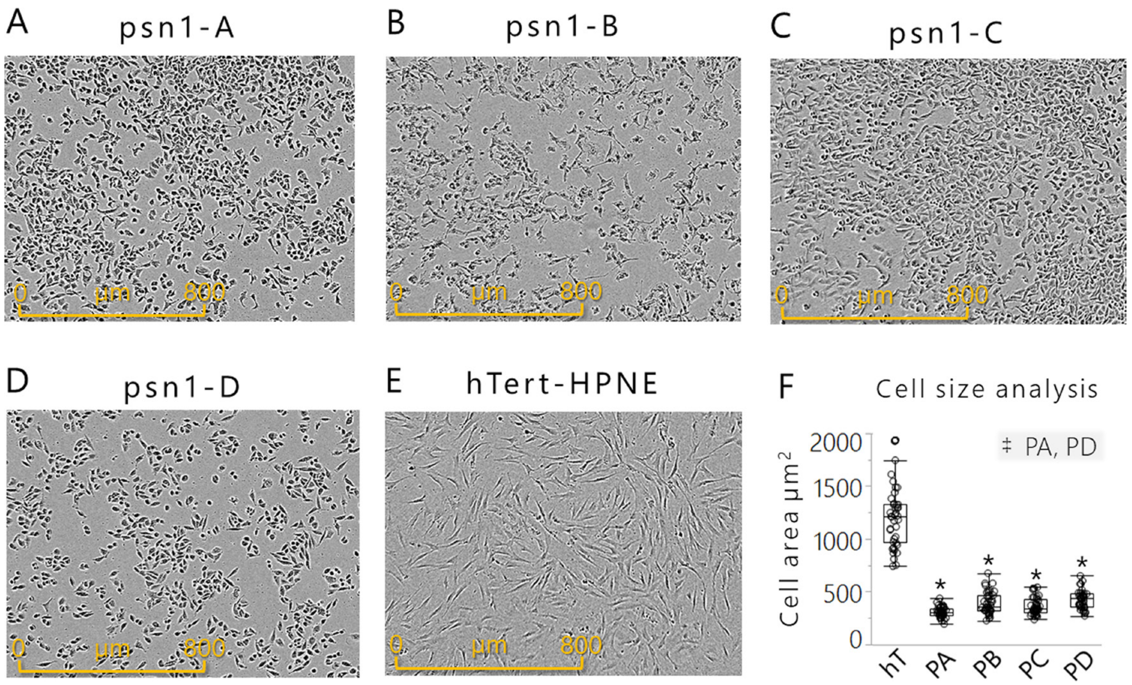

Supplementary Figure 3: Pancreatic cancer subclones and healthy control cells displayed variations in cell size and morphology. (**A-E**) Representative light microscope images of each cell group, including (A) psn1-A, (B) psn1-B, (C) psn1-C, (D) psn1-D, and (E) hTert cells. (F) Box plot of cell sizes measured by image analysis of healthy control (hT) and pancreatic cancer subculture groups. Data are represented as the cell area (μm^2) of 40 biological replicates per cell type. The Dunnett's Test was used to compare cancer groups to the healthy control, where *indicates *P* < 01. The Tukey-Kramer Test was used to determine significant differences between the cancer subcultures; the pair found to be significantly different is highlighted in the right-hand corner of the plot, where †indicates *P* = 0.0052.

A

Locus	Test Results *Healthy Control cells* Query Profile: hTert		ATCC Reference Database Profile: hTert-HPNE	
TH01	8	9	8	9
D5S818	11		11	
D13S317	12	13	12	13
D7S820	9	10	9	10
D16S539	12	13	12	13
CSF1PO	12		12	
Amelogenin	X	Y	X	Y
vWA	17		17	
TPOX	8	11	8	11
Total # of alleles in database profile	15			
# of shared alleles b/t query sample & database profile	15			
% match between sample & hTert-HPNE database profile	100%			

B

Locus	Test Results *Originating cells* Query Profile: PSN-1		Test Results *Subculture A* Query Profile: psn1-A		Test Results *Subculture B* Query Profile: psn1-B		Test Results *Subculture C* Query Profile: psn1-C		Test Results *Subculture D* Query Profile: psn1-D		ATCC Reference Database Profile: PSN-1	
TH01	6		6		6		6		6		6	
D5S818	11	13	11	13	11	13	11	13	11	13	11	13
D13S317	10		10		10		10		10		10	
D7S820	10		10		10		10		10		10	
D16S539	11		11		11		11		11		10	11
CSF1PO	12		12		12		12		12		12	
Amelogenin	X		X		X		X		X		X	
vWA	17		17		17		17		17		17	
TPOX	8	11	8	11	8	11	8	11	8	11	8	11
Total # of alleles in database profile	11		11		11		11		11			
# of shared alleles b/t query sample & database profile	12		12		12		12		12			
% match between sample & PSN-1 database profile	92%		92%		92%		92%		92%			

time=0 (Originating cells) — *Time~6 months* (Subcultures A–D)

Supplementary Figure 4: STR Profile Reports of healthy control and pancreatic cancer cells before and after subculturing experiment. (**A**) Results of STR profiling of healthy immortalized control cell line (hTert) relative to the ATCC reference database profile for hTert-HPNE. (**B**) Results of STR profiling of originating cell line at time=0 (PSN-1) as well as the four different isolated subculture groups (psn1-A, psn1-B, psn1-C, psn1-D) from the originating PSN-1 cell line after subculturing experiment, genotypic and phenotypic profiling (time~6 months) relative to the ATCC reference database profile of PSN-1.

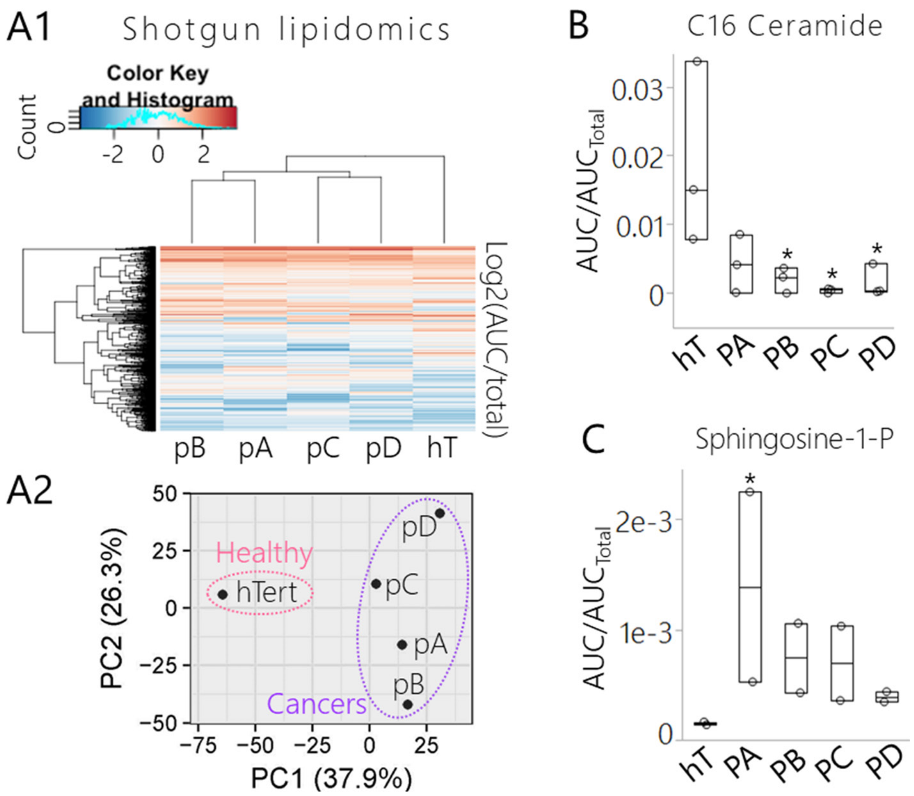

Supplementary Figure 5: Shotgun lipidomics suggests global shifts in lipid metabolism including conserved differences in pro-survival S1P and pro-apoptotic C16 Cer levels among pancreatic cancer subclones relative to healthy control cells. (**A1**–**A2**) Results of shotgun lipidomics analysis of pancreatic cancer subclone and healthy control cell lysates measured by direct-infusion ESI-MS. (A1) Hierarchal clustering and heat map of cancer subclones and healthy control lipidomes measured by direct-infusion ESI-MS. Rows were centered; no scaling was applied to rows. Both rows and columns were clustered using Hierarchal Euclidean distance metric with complete linkage. Each row represents a unique lipid annotation (*n* = 982 species). The color scale from -2 (blue) to 2 (orange) represents the normalized lipid concentration of one sample per group calculated for each lipid as Log2(AUC_{Lipid}/AUC_{Total}). (**A2**) PCA of cancer subclones and healthy control lipidomes measured by direct-infusion LC-MS. No scaling was applied to rows; SVD with imputation was used to calculate principal components; X and Y axis show principal component 1 and principal component 2 that explain 37.9% and 26.3% of the total variance, respectively. (**B**–**C**) Box plots depicting relative concentrations of (B) C16 Cer and (C) S1P that were detected in two biological replicates of samples from each of the pancreatic cancer group and the healthy control cell line via direct-infusion ESI-MS. (B–C) Data are depicted as the normalized concentration of each lipid (AUC_{Lipid}/AUC_{Total}). The Dunnett's test was used to determine significant differences between the cancer groups relative to the healthy control cell line, where *indicates $p < 0.05$.

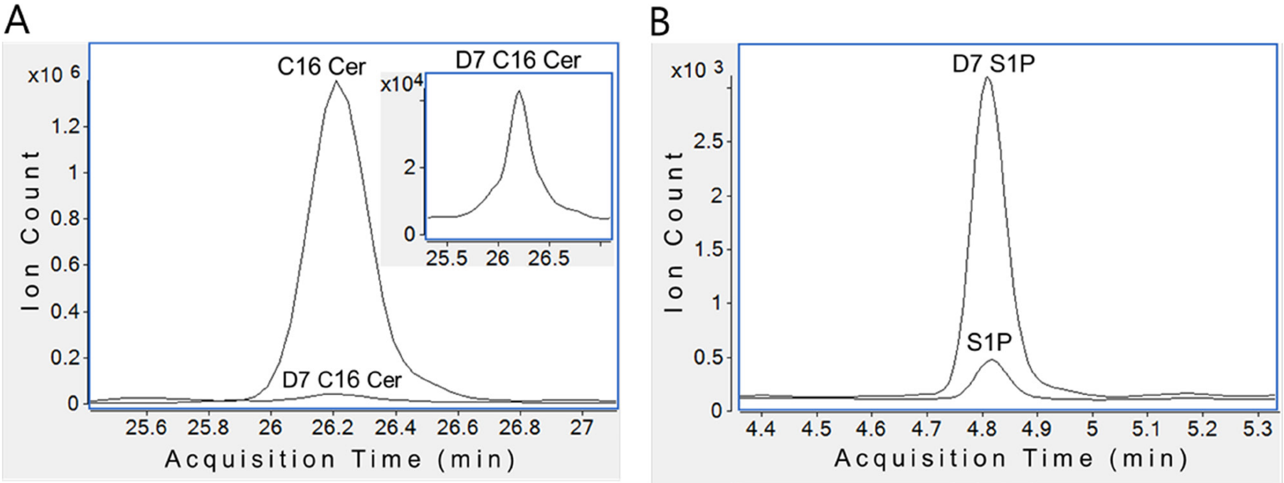

Supplementary Figure 6: Representative elution profiles of C16 Cer, S1P, and corresponding deuterated internal standards. (**A**) Representative elution profile of C16 Cer extracted from pancreatic cancer cell lysate spiked with C16 Cer-d7amide as the internal standard (inset is C16 Cer-d7 shown on a larger scale for clarity). The chromatogram shows ions for C16 Cer and C16 Cer-d7 both eluting at 26.2 minutes. The representative chromatogram was measured by LC-MS (QTOF) in lipid extract from pA cell lysate spiked with 50 pmol of C16 Cer-d7. The normalized C16 Cer concentration was calculated using the following equation: (50 $\text{pmol}_{\text{C16Cer-d7}}$) /($\text{AUC}_{\text{C16Cer-d7}}$) = ($x$ $\text{pmol}_{\text{C16Cer}}$)/($\text{AUC}_{\text{C16Cer}}$), where x = the normalized concentrations of C16 Cer. In cases where the signal:noise of the C17 Cer internal standard (not shown) was greater than C16 Cer-d7, C16 Cer was quantified by normalizing to C17 Cer instead of d7-C16 Cer. (**B**) Representative elution profile of S1P extracted from pancreatic cell lysate spiked with S1P-d7 as the internal standard. The chromatogram shows ions for S1P-d7 and S1P both eluting at 4.82 minutes. The representative chromatogram was measured by LC-MS (QqQ) in lipid extract from psn1-D (pD) cell lysate spiked with 100 pmol of S1P-d7. The area under the curve (AUC) for each compound was used to solve for S1P concentration: (100 $\text{pmol}_{\text{D7S1P}}$)/($\text{AUC}_{\text{D7S1P}}$) = ($x$ pmol_{S1P})/(AUC_{S1P}), where x = the normalized concentration of S1P.

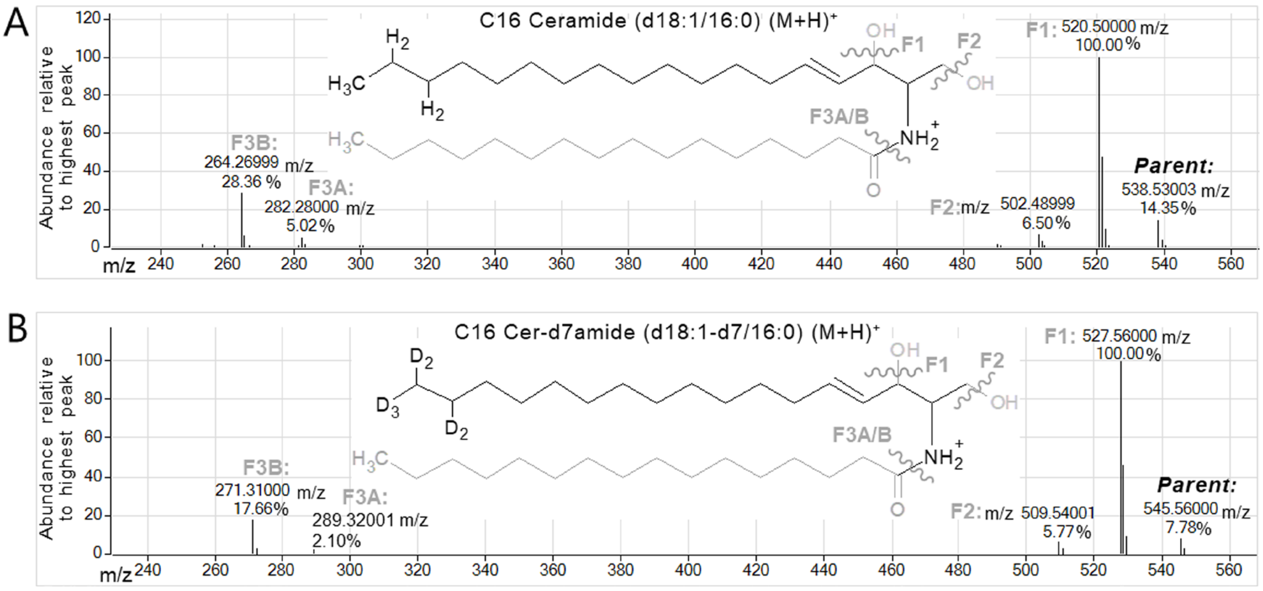

Supplementary Figure 7: Representative MS/MS fragmentation spectra of C16 Cer and corresponding internal standard C16 Cer-d7 measured by LC-MS of pancreatic cell lipids. (**A**) Representative MS/MS spectrum of C16 Cer Parent (precursor) ion 538.53 m/z, which represents $[C_{34}H_{67}NO_3+H]^+$, the protonated form of C16 Cer $(M + H)^+$ (note: the proton can be added to multiple sites on the molecule as it is subjected to electrospray ionization, only one possibility is shown). Fragment 1 (F1) 520.5 m/z is $[C_{34}H_{65}NO_2 + H]^+$ which represents the parent ion with one water loss $(M + H - H_2O)^+$, Fragment 2 (F2) 502.5 m/z is $[C_{34}H_{63}NO + H]^+$ which represents the parent ion with two water losses $(M + H - 2H_2O)^+$, Fragment 3A (F3A) 282.28 m/z is $[C_{18}H_{35}NO + H]^+$ which represents the parent ion that has lost one water molecule and the C16:0 fatty acyl chain (M + H - H_2O - fatty acyl), and Fragment 3B (F3B) 264.27 m/z is $[C_{18}H_{33}N + H]^+$ which represents the parent ion that has lost 2 water molecules and the acyl chain (M + H - $2H_2O$ - fatty acyl). (**B**) Representative MS/MS spectrum of C16 Cer-d7 Parent ion 545.56 is $[D_7C_{34}H_{60}NO_3 + H]^+$ which represents the protonated form of C16 Cer-d7 $(M + H)^+$; Fragment 1 (F1) 527.56 m/z is $[D_7C_{34}H_{58}NO_2 + H]^+$ which represents the parent ion with one water loss $(M + H - H_2O)^+$, Fragment 2 (F2) 509.54 m/z is $[D_7C_{34}H_{56}NO + H]^+$ which represents the parent ion with two water losses $(M + H - 2H_2O)^+$, Fragment 3A (F3A): 289.32 m/z is $[D_7C_{18}H_{28}NO + H]^+$ which represents the parent ion that has lost one water molecule and the C16:0 fatty acyl chain (M + H - H_2O, - fatty acyl), and Fragment 3B (F3B) 271.3 m/z is $[D_7C_{18}H_{26}N + H]^+$ which represents the parent ion that has lost 2 water molecules and the fatty acyl chain (M + H - $2H_2O$, - fatty acyl). The parent and each fragment of C16 Cer-d7 are 7 m/z units higher than the parent and corresponding fragments of C16 Cer, due to the presence of 7 deuterium atoms in place of 7 hydrogens on the 16:0 acyl tail of C16 Cer-d7. Because C16 Cer-d7 was spiked into each cell lysate prior to lipid extraction, the D7 fragmentation spectra allowed us to more accurately identify the naturally occurring C16 Cer in each cell sample. The representative spectra shown in (A–B) were collected from psn1-A (pA) cell lysate spiked with 50 pmol of C16 Cer-d7 (MS/MS Collision Energy = 8). The top number in each peak label is the m/z and the bottom number is the abundance normalized to highest peak in the spectra (F1). The grayed out portions of the chemical structures represent groups that have been removed from the parent ion in each fragment and the cleavage symbols suggest where bond breakage may have occurred in succession to form the indicated fragments.

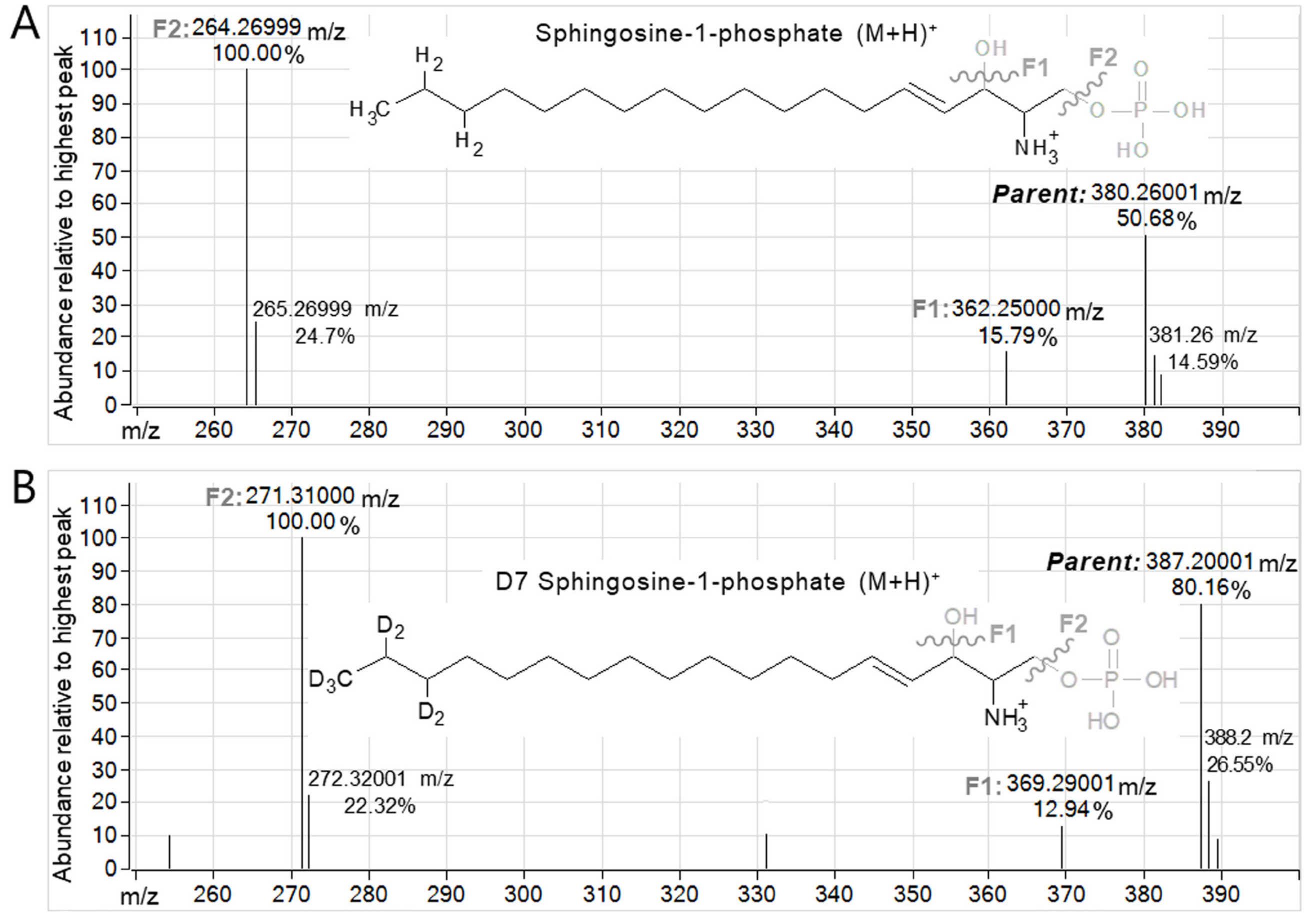

Supplementary Figure 8: Representative MS/MS fragmentation spectra of S1P and corresponding internal standard S1P-d7 measured by LC-MS of pancreatic cell lipids. (**A**) Representative MS/MS spectrum of S1P Parent (precursor) ion 380.26 m/z, which represents $[C_{18}H_{38}NO_5P + H]^+$, the protonated form of S1P $(M + H)^+$ (note: the proton can be added to multiple sites on the molecule as it is ionized in the mass spectrometer, one possibility is shown); Fragment 1 (F1) is 362.25 m/z is $[C_{18}H_{36}NO_4P + H]^+$ which represents the parent ion with a water loss $(M + H - H_2O)^+$ and Fragment 2 (F2) 264.27 m/z is $[C_{18}H_{34}N] + H]^+$ which represents the parent ion that has lost a water molecule and the phosphate group (M + H - H2O, - phosphate)$^+$. (**B**) Representative MS/MS spectrum of S1P-d7 Parent ion: 387.2 m/z, which is $[D_7C_{18}H_{31}NO_5P + H]^+$, the protonated form of S1P-d7 $(M + H)^+$, Fragment 1 (F1) 369.29 m/z is $[D_7C_{18}H_{29}NO_4P] + H]^+$ which represents the parent ion with a water loss (M + H - H2O)$^+$ and Fragment 2 (F2): 271.31 m/z is $[D_7C_{18}H_{27}N + H]^+$ which represents the parent ion that has lost a water molecule and the phosphate group $(M + H - H_2O, - \text{phosphate})^+$. Note that the parent and each fragment of S1P-d7 are 7 m/z units higher than the parent and corresponding fragments of S1P, due to the presence of the 7 deuterium atoms in place of 7 hydrogens on the acyl tail of S1P-d7. Because S1P-d7 was spiked into each cell lysate prior to lipid extraction, the D7 fragmentation spectra allowed us to more accurately identify the naturally occurring S1P in each cell-derived sample. The representative spectra were collected from psn1-A (pA) cell lysate spiked with 100 pmol of S1P-d7 (MS/MS Collision Energy = 8). The top number in each peak label is the respective m/z and bottom number is the abundance normalized to the highest peak in the spectra (F2). M0 and M1 isotopic peaks for the two most abundant in the spectra are also labeled (Parent and F2). The grayed out portions of the chemical structures represent groups that have been removed from the parent ion in each fragment and the cleavage symbols suggest where bond breakage successively occurred to form the indicated fragments.

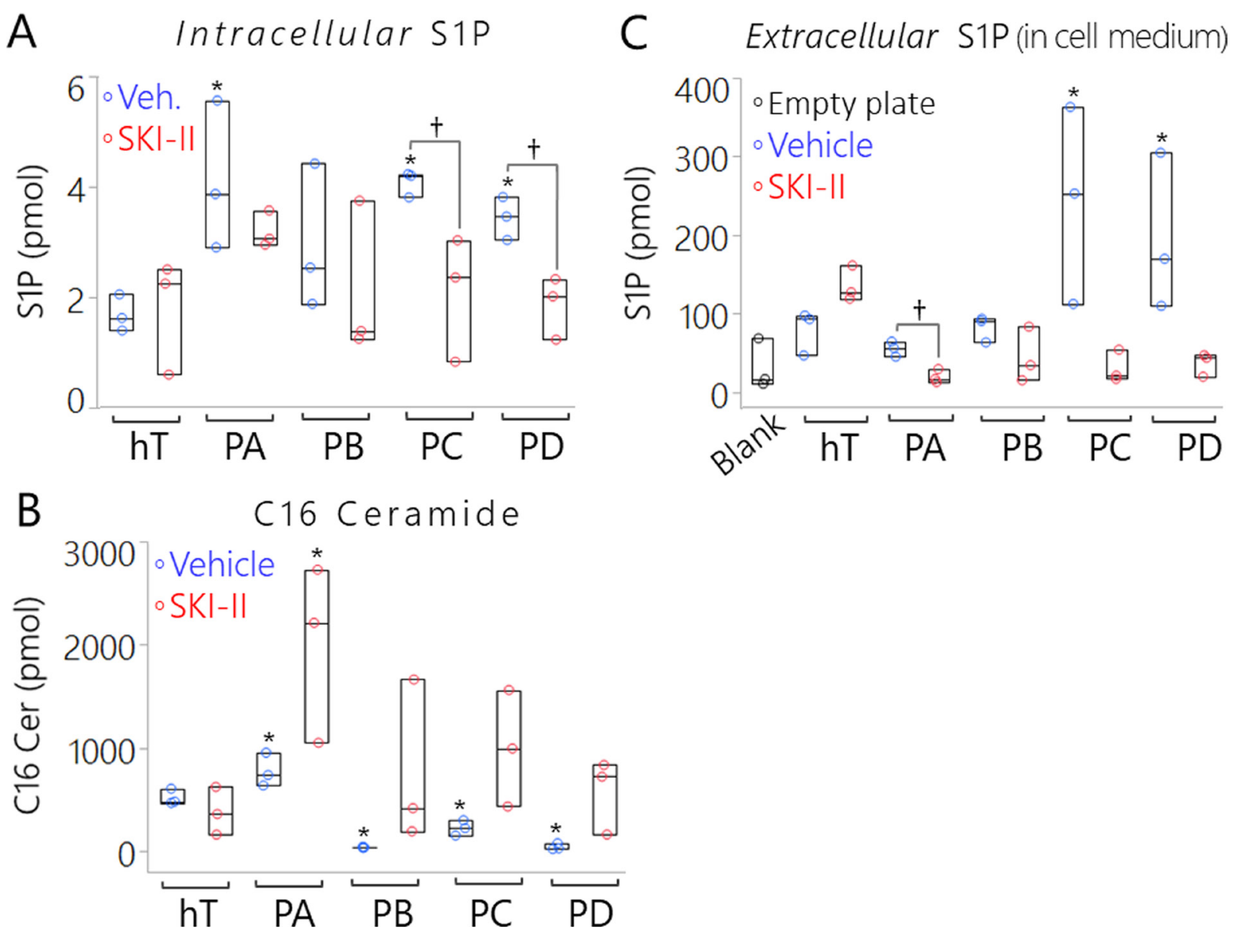

Supplementary Figure 9: S1P and C16 Cer levels were altered in pancreatic cancer subclones relative to the healthy control but may be corrected in part by SKI-II treatment. (**A**, **C**) Box plots of S1P concentrations measured by LC-MS of lipids extracts from (A) whole cell lysates and (C) complete cell medium from corresponding cell culture dishes after treatment with the Vehicle (1x PBS) (blue circles) or SKI-II (13 µM) (red circles) for 12 hours. Note that the "Blank" in (C) represents purely FBS-derived S1P levels measured in fresh complete cell medium (RPMI 1640 + 10% FBS) that was never exposed to cell cultures. (A, C) Data were collected in biological triplicate and represented as the AUC of S1P normalized to the S1P-d7 internal standard. (B) Box plot of C16 Cer concentrations measured by LC-MS of the same samples depicted in (A). Data are represented as the AUC of C16 Cer normalized to the C16 Cer-d7 or C17 Cer internal standard. (A–C) The Dunnett's Test was used to compare the lipid levels measured in the cancer groups to the healthy control under the same treatment (Vehicle or SKI-II) where *indicates $P < 0.05$. Student's t-tests were used to compare the SKI-II treated group of each cell type to the respective Vehicle Control group, wherein †indicates $P < 0.05$.

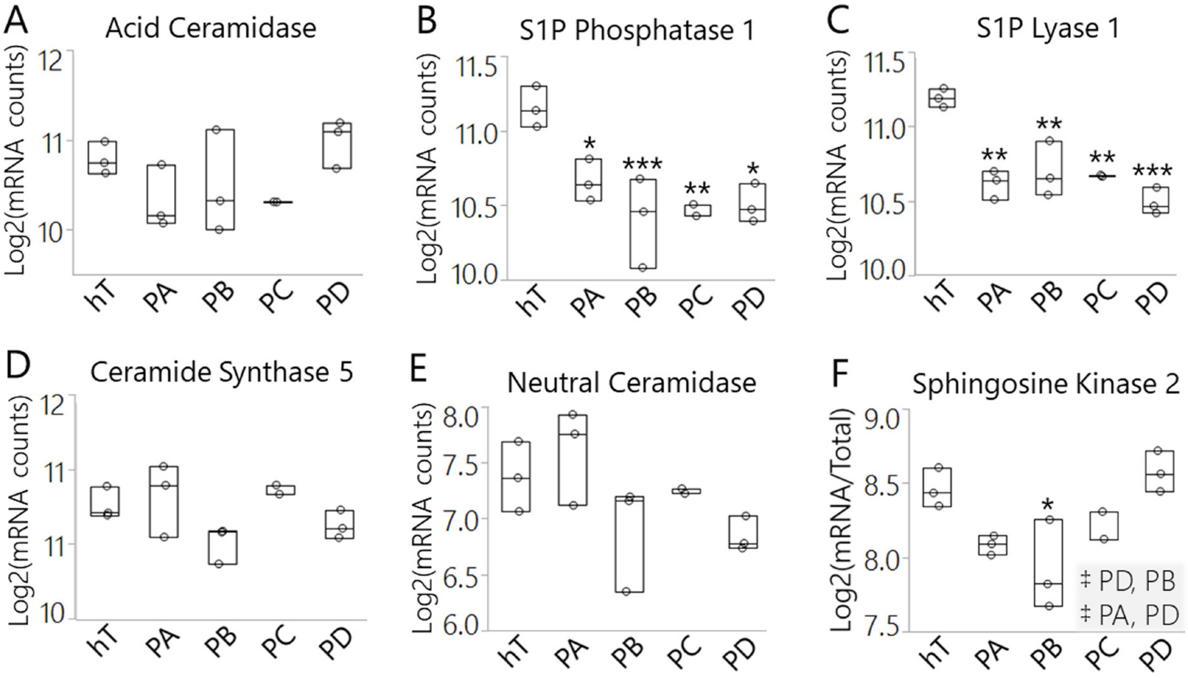

Supplementary Figure 10: Comparison of mRNA expression of enzymes directly involved in S1P/C16 Cer metabolic pathway. (**A**–**F**) Box plots of normalized mRNA levels of sphingolipid-modifying enzymes measured by RNA-Seq of pancreatic cancer subcultures (pA, pB, pC, pD) and healthy control cells (hT), including (A) Acid Ceramidase, (B) S1P phosphatase 1, (C) S1P Lyase 1, (D)) Ceramide Synthase 5, (E) Neutral Ceramidase, and (F) Sphingosine Kinase 2. (A–F) Measurements were collected in biological triplicate and expressed on a Log2 scale. The Dunnett's test was used to determine differences between mRNA levels in the cancer groups relative to the healthy control; the ***indicates $P \leq 01$, **indicates $P < 0.001$, and *indicates $P < 0.05$. Tukey-Kramer Tests were used to determine significant differences between cancer subcultures; pairs that were significantly different are highlighted in the comma-separated list on the right-hand corner of (F), where ‡indicates $P < 0.05$.

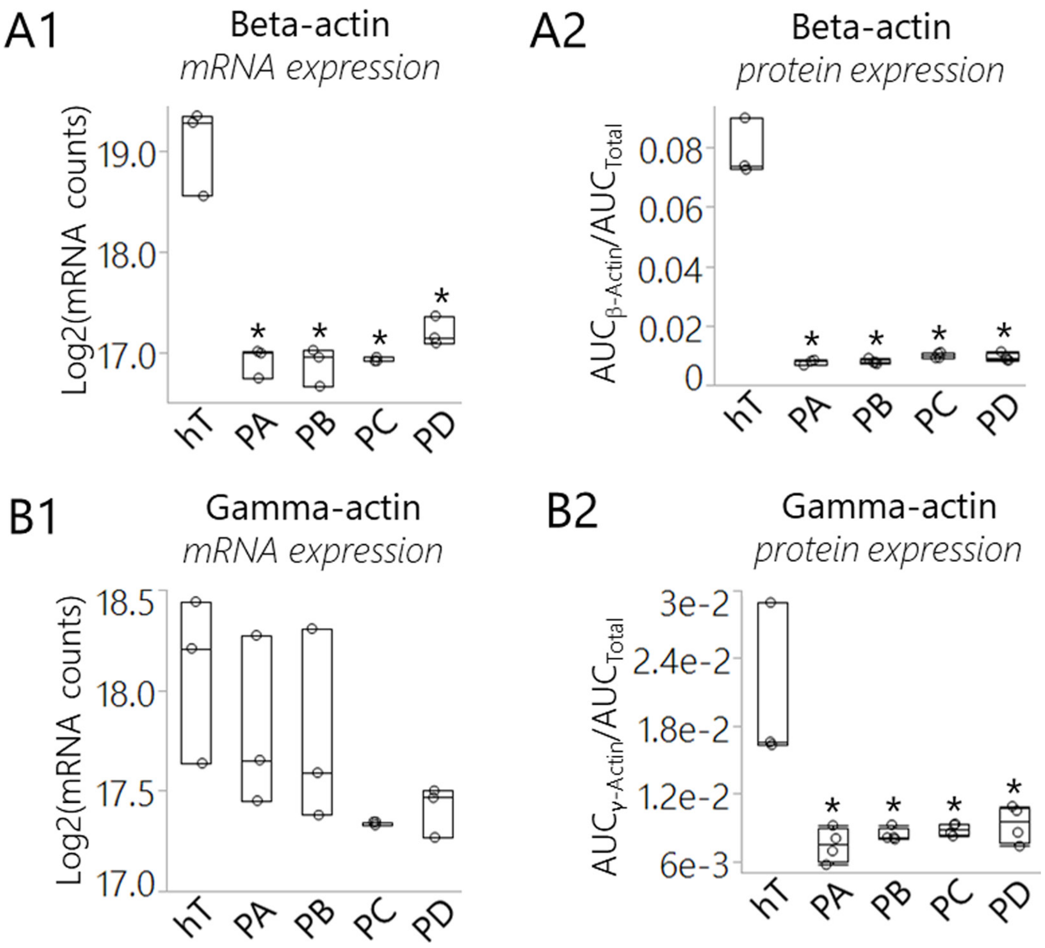

Supplementary Figure 11: Cytoplasmic actin mRNA and protein levels were reduced in pancreatic cancer subclones relative to the healthy control. (**A1, B1**) Box plots of mRNA expression levels of (A1) Cytoplasmic Actin 1 (Beta-actin) and (B1) Cytoplasmic Actin 2 (Gamma-actin) measured in biological triplicate in pancreatic cancer subclones (pA, pB, pC, pD) and healthy control cells. Data are represented as the normalized mRNA counts on a Log2 scale. (**A2**, **B2**) Box plots of normalized protein concentrations of (**A2**) Cytoplasmic Actin 1 (Beta-actin) and (**B2**) Cytoplasmic Actin 2 (Gamma-actin) measured in biological triplicate (hT) or quadruplet (pA-pD). (A2, B2) Data are represented as the mean normalized protein concentration in each sample measured in biological triplicate. The Dunnett's Test was used to compare the cancer cell groups to the healthy control; the *indicates $p < 01$. Based on the Tukey-Kramer Test, there were no significant differences between mRNA or protein levels of actin measured in the cancer groups.

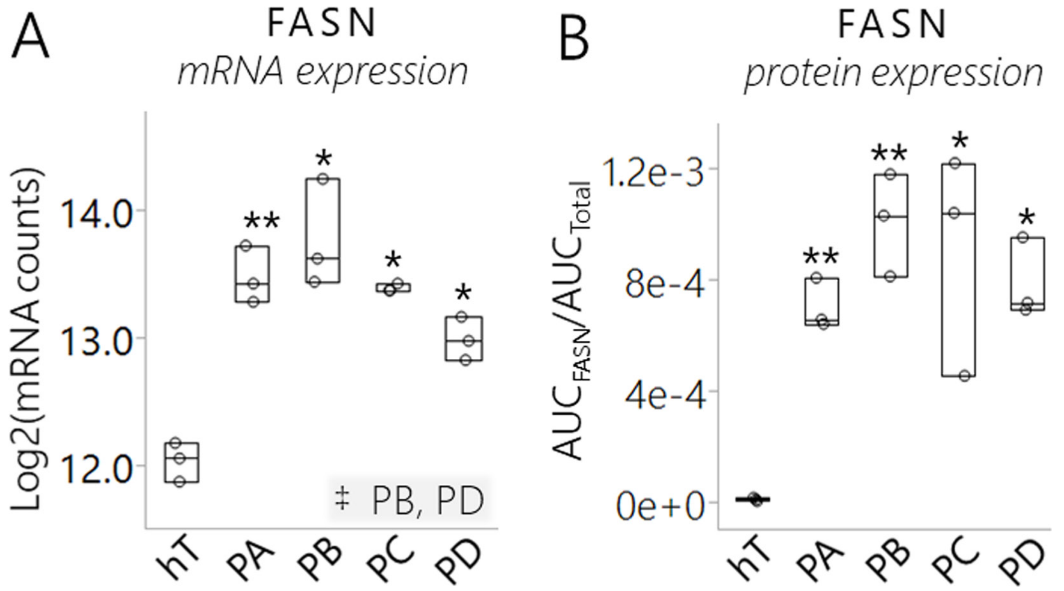

Supplementary Figure 12: Fatty Acid Synthase mRNA and protein levels were increased in pancreatic cancer subclones relative to the healthy control. (**A**, **B**) Box plots of (A) mRNA and (B) protein levels of Fatty Acid Synthase (FASN) measured in pancreatic cancer cell groups and the healthy control. (A) Data are represented as the normalized mRNA counts measured in biological triplicate in each cell type. (B) Data are represented as the normalized protein concentration measured in biological triplicate. (A, B) The Dunnett's Test was used to compare the cancer cell groups to the healthy control, where **indicates $P < 0.01$ and *indicates $p \leq 0.002$. Tukey-Kramer Tests were used to determine significant differences between the cancer groups; the pair found to be significantly different is highlighted in the right-hand corner of (A), where †indicates $P < 0.05$.

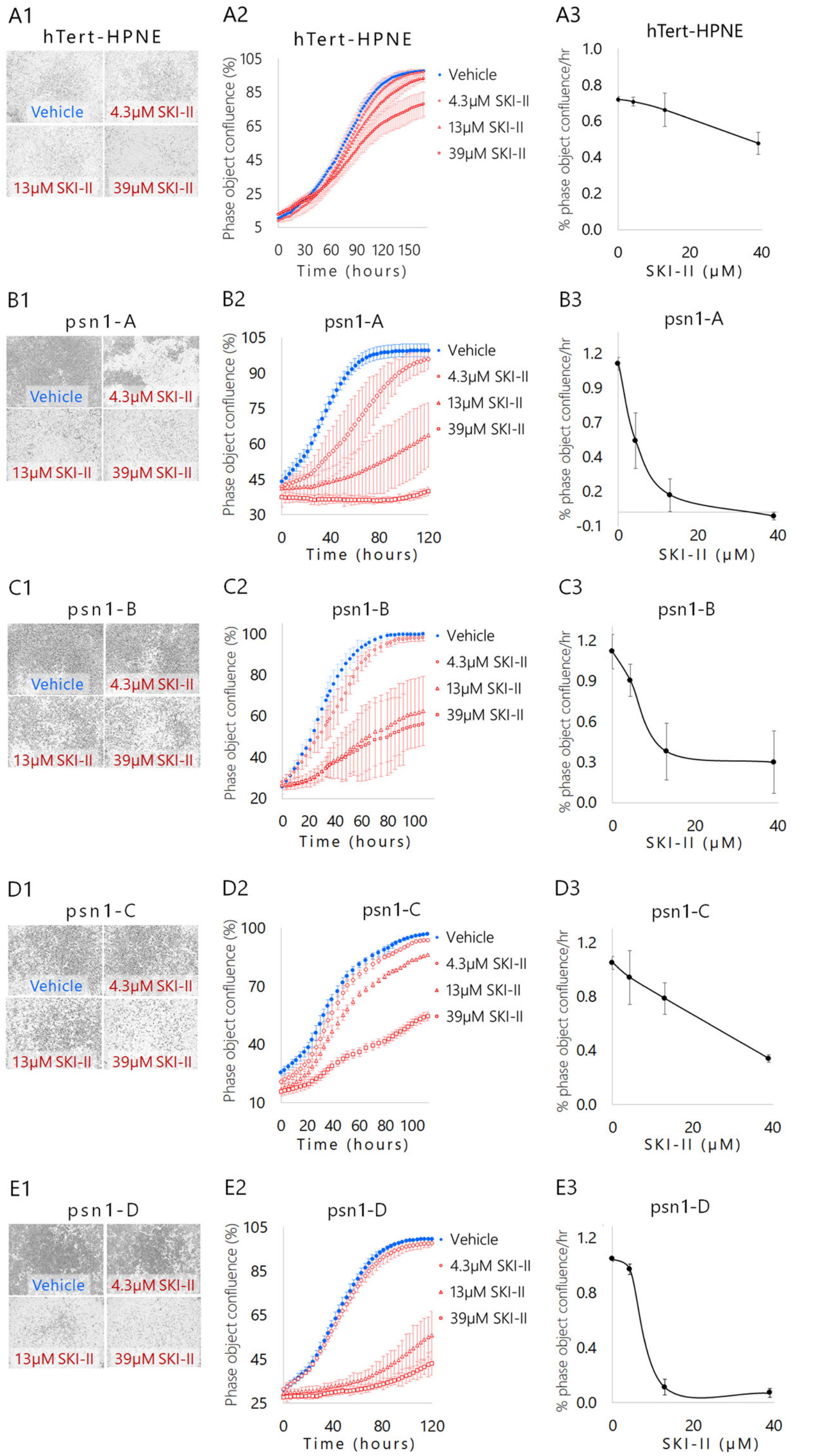

Supplementary Figure 13: Results of growth experiments of pancreatic cancer subclones and healthy control cells treated with increasing concentrations of SKI-II. (**A1**, **B1**, **C1**, **D1**, **E1**) Representative confluence images of (**A1**) hT, (**B1**) pA, (**C1**), pB, (**D1**) pC, and (**E1**) pD cells after 72 hours of treatment with the Vehicle (1x PBS) (top left), 4.3 µM SKI-II (top right), 13 µM SKI-II (bottom left), and 39 µM SKI-II (bottom right). (A–E) Representative growth curves of (A2) hT, (B2) pA, (C2), pB, (D2) pC, and (E2) pD cells during treatment with the Vehicle (blue points), 4.3 µM SKI-II (red circles), 13 µM SKI-II (red triangles), and 39 µM SKI-II (red squares). (A2, B2, C2, D2, E2) Data are represented as the mean phase object confluence ± SEM of four biological replicates over time (hours). (A3, B3, C3, D3, E3) Dose-dependent effects of SKI-II on the growth rates of (A3) hT, (B3) pA, (C3), pB, (D3) pC, and (E3) pD cells. Growth rates were determined by calculating the slope of the linear-like growth phase of each cell type, where x = time (hours) and y = percent phase object confluence. Data are represented as the mean proliferation rate ± SEM of four biological replicates per group plotted against the SKI-II concentration used to treat each sample (µM).

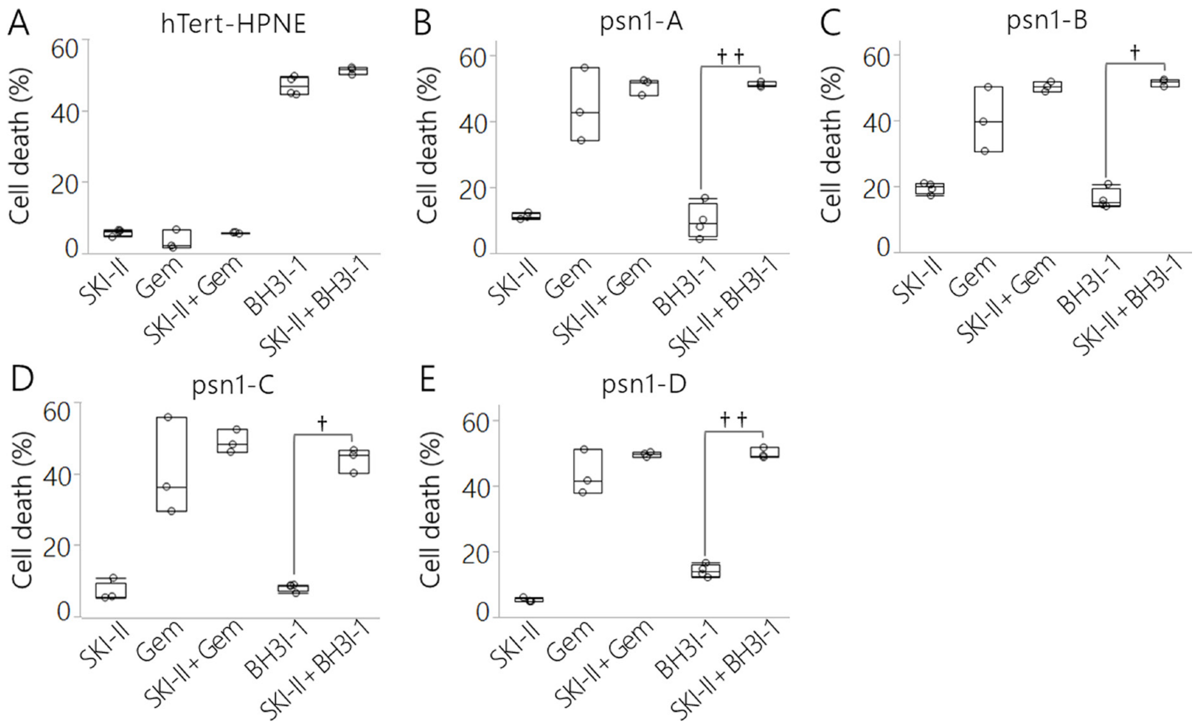

Supplementary Figure 14: SKI-II treatment significantly sensitized pancreatic cancer subclones to mitochondria-mediated apoptotic signals. (**A**–**F**) Results of Cell Death Assay of cancer subclones (pA, pB, pC, and pD) and hT cells treated with the following drug combinations: (1) SKI-II only, (2) control apoptosis inducer Gemcitabine (Gem) only, (3) SKI-II combined with Gem, (4) mitochondria-mediated apoptosis inducer BH3I-1, and (5) SKI-II combined with BH3I-1 for 12 hours at the following concentrations based on the combinatorial EC_{50} of each group: (A) hTert: (1) 13 μM SKI-II, (2) 10 μM Gem, (3) 13 μM SKI-II + 10 μM Gem, (4) 1 μM BH3I-1, and (5) 13 μM SKI-II + 1 μM BH3I-1; (B) psn1-A: (1) 1 μM SKI-II, (2) 9 μM Gem, (3) 1 μM SKI-II + 9 μM Gem, (4) 7 μM BH3I-1, and (5) 1 μM SKI-II + 7 μM BH3I-1; (C) psn1-B: (1) 2 μM SKI-II, (2) 9 μM Gem, (3) 3 μM SKI-II + 9 μM Gem, (4) 8 μM BH3I-1, and (5) 2 μM SKI-II + 8 μM BH3I-1; (D) psn1-C: (1) 8 μM SKI-II, (2) 5 μM Gem, (3) 6 μM SKI-II + 5 μM Gem, (4) 4 μM BH3I-1, and (5) 8 μM SKI-II + 4 μM BH3I-1; (E) psn1-D: (1) 6 μM SKI-II, (2) 5 μM Gem, (3) 4 μM SKI-II + 5 μM Gem, (4) 3 μM BH3I-1, and (5) 6 μM SKI-II + 3 μM BH3I-1. (A–E) After the 12 hour treatment, dead cells were stained with propidium iodide and quantified by flow cytometry. Samples were measured in biological triplicate and depicted as percent cell death normalized to vehicle controls. Student's t-tests were used to compare between cell death responses of groups treated with an apoptosis inducer alone (Gem or BH3I-1) versus those treated with an apoptosis inducer combined with SKI-II, wherein †indicates $P < 0.0001$ and $^{\dagger}P \leq 0.001$.

Supplementary Dataset 1 : (A) Genome-wide nscSNPs per Gene. See Supplementary_Dataset_1

Supplementary Dataset 2: (B) RNA-Seq differentially expressed genes all groups. See Supplementary_Dataset_2

Supplementary Dataset 3: (C) RNA-Seq differentially expressed genes cancer clones. See Supplementary_Dataset_3

Supplementary Dataset 4: (D) Quantitative global proteomics. See Supplementary_Dataset_4

Supplementary Dataset 5: (E) Proteomics differentially expressed proteins and ontologies hT vs. clones. See Supplementary_Dataset_5

Supplementary Dataset 6: (F) Proteomics differentially expressed proteins and ontologies pA vs. other clones. See Supplementary_Dataset_6

Supplementary Dataset 7: (G) Proteomics differentially expressed proteins and ontologies pB vs. other clones. See Supplementary_Dataset_7

Supplementary Dataset 8: (H) Proteomics differentially expressed proteins and ontologies pC vs. other clones. See Supplementary_Dataset_8

Supplementary Dataset 9: (I) Proteomics differentially expressed proteins and ontologies pD vs. other clones. See Supplementary_Dataset_9

Supplementary Dataset 10: (J) Shotgun quantitiative lipidomics. See Supplementary_Dataset_10

Supplementary Dataset 11: (K) LC-MS-QTOF Intracellular lipids RTs and Quant. See Supplementary_Dataset_11

Supplementary Dataset 12: (L) LC-MS-QTOF Extracellular lipids RTs and Quant. See Supplementary_Dataset_12

Supplementary Dataset 13: (M) nscSNP Analysis sphingolipid pathway proteins. See Supplementary_Dataset_13

Supplementary Dataset 14: (N) Targeted lipidomics RTs and Quant. See Supplementary_Dataset_14

Supplementary Dataset 15: (O) LC-MS-QTOF FBS Batch test lipids RTs and Quant. See Supplementary_Dataset_15
